# Prevalent intron retention fine‐tunes gene expression and contributes to cellular senescence

**DOI:** 10.1111/acel.13276

**Published:** 2020-12-04

**Authors:** Jun Yao, Dong Ding, Xueping Li, Ting Shen, Haihui Fu, Hua Zhong, Gang Wei, Ting Ni

**Affiliations:** ^1^ State Key Laboratory of Genetic Engineering Collaborative Innovation Center of Genetics and Development Human Phenome Institute School of Life Sciences Fudan University Shanghai P.R. China; ^2^ Department of Population Health NYU Langone School of Medicine New York NY USA

**Keywords:** CPNE1, intron retention, senescence, splicing factor, U2AF1

## Abstract

Intron retention (IR) is the least well‐understood alternative splicing type in animals, and its prevalence and function in physiological and pathological processes have long been underestimated. Cellular senescence contributes to individual aging and age‐related diseases and can also serve as an important cancer prevention mechanism. Dynamic IR events have been observed in senescence models and aged tissues; however, whether and how IR impacts senescence remain unclear. Through analyzing polyA^+^ RNA‐seq data from human replicative senescence models, we found IR was prevalent and dynamically regulated during senescence and IR changes negatively correlated with expression alteration of corresponding genes. We discovered that knocking down (KD) splicing factor U2AF1, which showed higher binding density to retained introns and decreased expression during senescence, led to senescence‐associated phenotypes and global IR changes. Intriguingly, *U2AF1*‐KD‐induced IR changes also negatively correlated with gene expression. Furthermore, we demonstrated that U2AF1‐mediated IR of specific gene (*CPNE1* as an example) contributed to cellular senescence. Decreased expression of *U2AF1*, higher IR of *CPNE1*, and reduced expression of *CPNE1* were also discovered in dermal fibroblasts with age. We discovered prevalent IR could fine‐tune gene expression and contribute to senescence‐associated phenotypes, largely extending the biological significance of IR.

## Graphical abstract

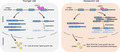

## 1 INTRODUCTION

Cellular senescence is defined as a process that cells enter to the state of irreversible cell‐cycle arrest after serial proliferation (Hayflick & Moorhead, 1961; Lopez‐Otin et al., 2013). Senescent cells are distinct from young ones by several morphological and molecular markers, including flattened and enlarged cell morphology, increased senescence‐associated β‐galactosidase (SA‐β‐Gal) activity, reduced proliferation rate, the absence of proliferative marker MKI67, elevated expression of DNA damage markers, tumor suppressors, and cell‐cycle inhibitors (p16, p21, p15, and p27) (Dimri et al., 1995; Munoz‐Espin & Serrano, 2014). Cell senescence has been regarded as one of the key hallmarks of aging and an important contributor to aging and age‐related diseases (Lopez‐Otin et al., 2013; Munoz‐Espin & Serrano, 2014). In addition, it has been reported that cell senescence involved in embryonic development (Munoz‐Espin et al., 2013; Storer et al., 2013), tissue degeneration (Munoz‐Espin & Serrano, 2014; Sturmlechner et al., 2017; Van Deursen, 2014), and cancer prevention (Serrano et al., 1997). Studies have shown that senescent cell number progressively increases in the tissues/organs of aged individuals (Jeyapalan et al., 2007), and removal of senescent cells can extend healthy life span in mice (Baker et al., 2016). These findings illustrate the biological importance of cellular senescence.

Transcriptomic and/or proteomic changes have been reported to be associated with phenotypic alterations of senescent cells (Kim et al., 2013; Mazin et al., 2013; Waldera‐Lupa et al., 2014; Wei et al., 2015), and stage‐specific gene expression modules can reflect the progressive senescence phenotypes (Kim et al., 2013). Therefore, it is crucial to understand the underlying gene expression regulation to illuminate the mechanism of cellular senescence. Alternative splicing (AS) is a crucial step in eukaryotic gene expression, and widespread AS changes have been discovered in both senescence and aging process (Deschênes & Chabot, 2017). Impaired splicing caused by a de novo point mutation in *LMNA* was found to generate progerin and thus contributed to premature aging phenotypes in mice (Rodríguez et al., 2016). In addition, deregulation of AS was also associated with human peripheral blood leukocyte aging (Harries et al., 2011), human brain aging (Mazin et al., 2013), and even Alzheimer's disease susceptibility (Raj et al., 2018). These studies demonstrate the significance of AS in aging/senescence research.

Intron retention (IR), one of the basic AS modes, is a widespread post‐transcriptional mechanism regulating gene expression, which contributes to transcriptome diversity and influences various biological processes (Schmitz et al., 2017). Genome‐wide analysis had revealed prevalence of IR across multiple cell and tissue types of human and mouse (Braunschweig et al., 2014). IR has been reported to progressively accumulate during differentiation, development, and terminal erythropoiesis (Braunschweig et al., 2014; Pimentel et al., 2016). Besides, widespread IR characterizes many cancer transcriptomes, with the potential to inactivate tumor suppressor genes (Dvinge & Bradley, 2015; Jung‐ et al., 2015). IR was the major form of AS found in hypoxic tumor cells (Memon et al., 2016). Moreover, IR can also serve as a new source of neoepitopes in cancer (Smart et al., 2018). Interestingly, elevated IR was recently discovered to be a molecular signature during aging and also associated with Alzheimer's disease (Adusumalli et al., 2019). One possible function of IR is to downregulate steady‐state gene expression by either nuclear degradation machinery (Yap et al., 2012) or cytoplasmic nonsense‐mediated decay (NMD) (Wong et al., 2013). RNA‐binding proteins (RBPs), especially splicing factors, have been demonstrated to be involved in IR regulation (Cho et al., 2014; Wong et al., 2017; Yap et al., 2012). Decreased expression of splicing factors such as SNMP40 and SF3B1 is associated with IR in granulopoiesis (Wong et al., 2013). Many RBPs showed dynamic expression changes in aging tissues and some correlated well with cellular senescence (Holly et al., 2013; Masuda et al., 2012). Intriguingly, in senescent endothelial cells, IR of the endoglin‐coding gene *ENG* was regulated by SRSF1 (or ASF/SF2) and correlated with senescence‐associated phenotypes (Blanco & Bernabeu, 2011, 2012; Blanco et al., 2008). However, whether dynamic changes of IR play a causal role to senescence and which RBP regulates global IR and senescence remain elusive.

To address these two interesting questions, we started with systematic bioinformatical analysis on time‐series RNA‐seq data derived from two in vitro replicative senescence models (Marthandan et al., 2015), resulting in hundreds of progressively changed IR events during senescence. By combining genomics analyses and candidate gene approach, we proved that IR could play a causal role in regulating cellular senescence, and the splicing factor U2AF1 could regulate IR and, in turn, senescence. Furthermore, upregulated IR in a novel target gene of U2AF1, *CPNE1*, was discovered to induce senescence.

## 2 RESULTS

### 2.1 IR is prevalent during cellular senescence

**FIGURE 1:**
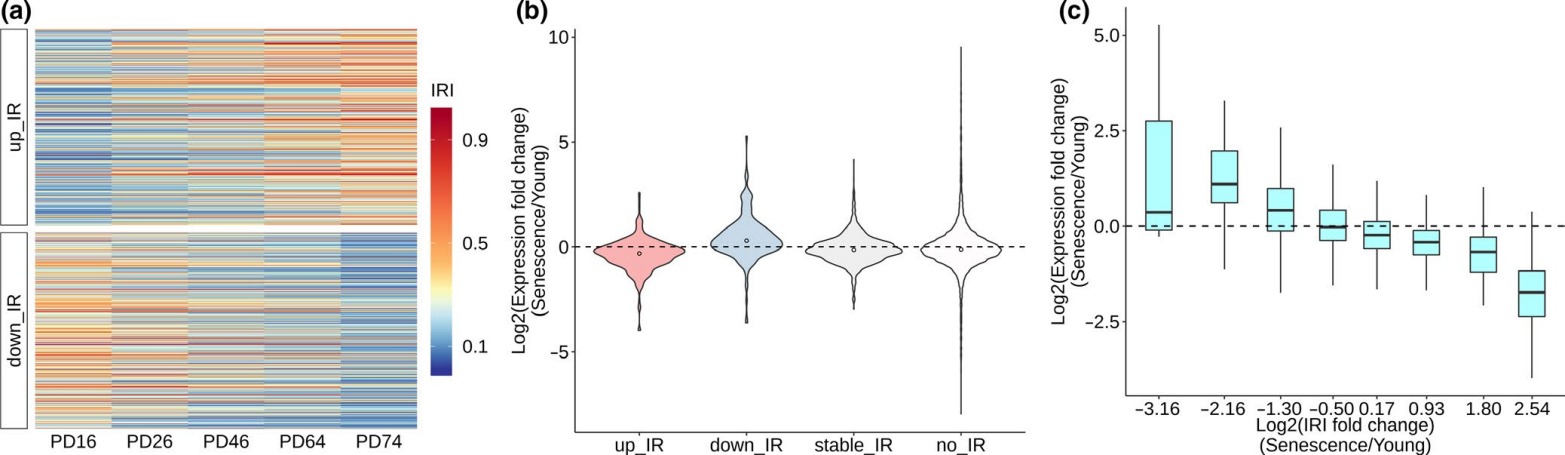
IR changes negatively correlated with gene expression during HFF senescence. (a) Heatmap showing IRI continuously up‐ and downregulated (up_IR and down_IR) during HFF cellular senescence. PD: population doubling. (b) Violin plot showing the distribution of gene expression changes between senescent (PD74) and young (PD16) HFF cells for genes of different IR groups. (c) Box plot showing the negative correlation between IR changes and corresponding gene expression alterations. Genes were divided into 8 bins based on IRI fold changes between HFF PD74 and PD16 (x‐axis)

To investigate whether global IR exists in cellular senescence, we first used published polyA^+^ RNA‐sequencing (RNA‐seq) data derived from human foreskin fibroblast (HFF) consisting of five different time points, each has three biological replicates (Marthandan et al., 2015). Intron retention index (IRI) (Ni et al., 2016) was adopted to quantify and evaluate the retention level for each intron (see “EXPERIMENTAL PROCEDURES” for details). As gene body coverage of sample PD46_3 showed a 3ʹ bias (Figure S1), it was discarded in downstream analysis to reliably calculate IRI. With an IRI threshold of 0.1 in at least one time point, retained introns were discovered in about one thirds (3,180/9,040) of the expressed genes (see Table S1 for a complete summary of IRI values) and most IR‐containing genes harbor only one IR events (Figure S2). We next classified introns into four groups for downstream analysis based on the IRI changes along senescence: continuous up (up_IR), continuous down (down_IR), no significant changes (stable_IR), and spliced intron (no_IR) (see details in “EXPERIMENTAL PROCEDURES”). We found 882 IR events showing continuously increased (up_IR, including 443 introns belong to 368 genes) or decreased (down_IR, including 439 introns belong to 333 genes) IRI in HFF cells along the five time points (Figure 1a). These continuously changed IR (up_IR +down_IR) were probably regulated by some factors important for senescence, and we thus defined them as regulated IR (change_IR, in 685 genes). Interestingly, we also found hundreds of such regulated IR events in another replicative senescence model, lung embryonal fibroblast (MRC‐5) (Figure S3a). To confirm the reliability of these IR events, genomic features such as intron length, GC content, and splicing strength were analyzed (Figure S4) and the features resembled those found in other biological processes (Amit et al., 2012; Lim & Burge, 2001; Sakabe & De Souza, 2007). Moreover, the same analysis on polyA^+^ RNA‐seq data of MRC‐5 cells reached similar results (Figure S5). These results indicate the prevalence of IR during cellular senescence.

### 2.2 IR changes negatively correlate with gene expression alterations during senescence

We speculated that dynamic changes of IR during senescence may fine‐tune gene expression, given retained introns tend to generate unstable transcripts that are usually degraded by cellular RNA surveillance machinery (Wong et al., 2013; Yap et al., 2012). In line with the higher degradation rate of IR transcripts compared with the spliced ones, genes with IR_up showed decreased expression trend while those with IR_down displayed increased expression trend in senescent cells compared with younger ones (Figure 1b, and Figure S3b, including both HFF and MRC‐5). As internal controls, genes with stable_IR and no_IR exhibited no obvious bias in expression change (Figure 1b, Figure S3b). Further analysis also revealed that IRI increase degree was linearly correlated with the extent of decreased gene expression in both HFF and MRC‐5 cells (Figure 1c, Figure S3c). These results indicate that altered IR negatively correlates with changes of gene expression during senescence. We further found that 67 out of 685 IR regulated genes were reported to be senescence‐associated archived in Human Cellular Senescence Gene Database (HCSGD) (Dong et al., 2017) (Figure S6). Some of these genes were even experimentally validated as senescence‐causing genes (e.g., *SIRT3* (Brown et al., 2013), *SIRT6* (Mao et al., 2012; Wu et al., 2015), and *RECQL4* (Croteau et al., 2012)). These findings suggest that genes with regulated IR have links to senescence.

### 2.3 Regulated introns during senescence have distinct RBP‐binding features

We next asked why certain introns were retained and why some retained introns changed dynamically while others remained unchanged. Differences in sequence and RBP‐binding property of related introns may provide clues to explain the IR regulation (Cho et al., 2014; Sakabe & De Souza, 2007). Change_IR tended to have significantly higher GC content (Figure S7, *p* = 0.026, one‐sided Wilcoxon's test), shorter length (Figure S8, *p* = 0.017, one‐sided Wilcoxon's test) and weaker 3ʹ splicing strength (Figure S9, *p* = 0.032, two‐sided Wilcoxon's test) compared to stable_IR, but no significant difference in 5ʹ splicing strength existed between change_IR and stable_IR (Figure S9). We next analyzed intron‐located‐binding sites of 171 RBPs that recorded in POSTAR2 database, which collected experimentally identified binding sites of RBPs based on diverse CLIP (crosslinking and immunoprecipitation of RNA–protein complexes) methods (HIST‐CLIP, iCLIP, PAR‐CLIP, and eCLIP) (Hu et al., 2017). The result showed that regulated introns (change_IR) and constitutively retained introns (stable_IR) both had higher RBP‐binding density than spliced introns (no_IR) (Figure S10). In addition, RBP‐binding density was significantly lower in change_IR than stable_IR, implying that the splicing efficiency of change_IR is more sensitive to RBP expression changes during senescence.

### 2.4 RNA‐binding protein U2AF1 downregulation induces widespread intron retention and cellular senescence

**FIGURE 2:**
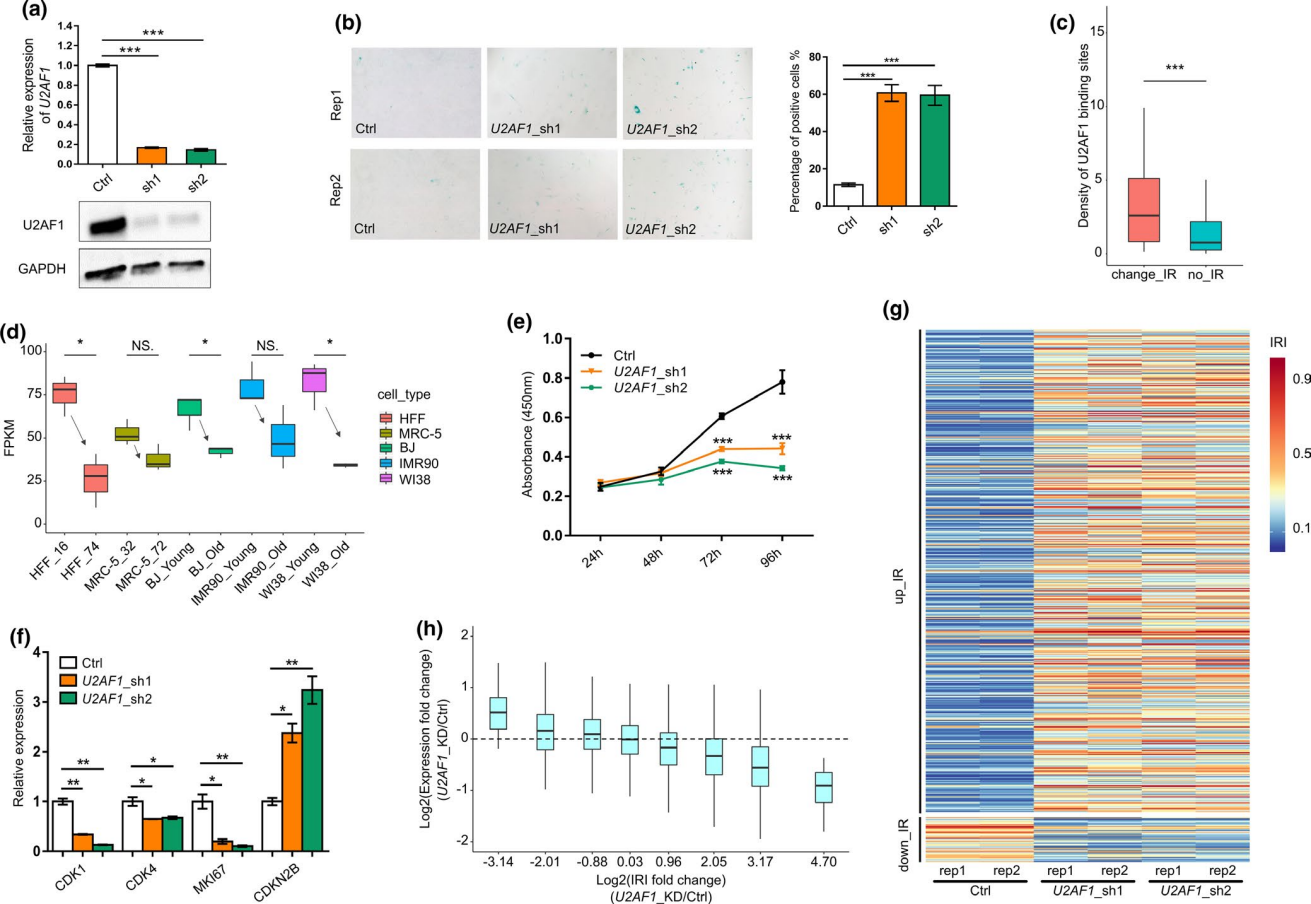
Decreased U2AF1 level causes senescence and IR in primary HFF cells. (a) qRT‐PCR (upper panel) and Western blot (lower panel) to validate *U2AF1* knockdown by two shRNAs (sh1 and sh2) in HFF cells. (b) SA‐β‐Gal staining of *U2AF1* knockdown and control (Ctrl) HFF cells. Bar chart shows the percentage of SA‐β‐Gal‐positive staining cells. (c) Box plot showing U2AF1‐binding density higher in regulated introns (change_IR) than spliced introns (no_IR) in HFF cells. *** indicates *p* < 0.001, one‐sided Wilcoxon's test. (d) Box plot showing decreased U2AF1 expression trend based on RNA‐seq data (three replicates for each sample) in five human replicative senescence models. (e) Proliferation rate of *U2AF1*‐KD and control HFF cells measured by CCK‐8 assay. (f) Gene expression evaluation of *CDK1*, *CDK4*, *MKI67*, and *CDKN2B* in *U2AF1*‐KD and control cells by qRT‐PCR. (g) Heatmap showing global IR changes upon knockdown of U2AF1. Rep1 and rep2 represent two biological replicates for each shRNA. (h) Box plot showing the degree of IR changes negatively correlates with the extent of expression fold changes. Genes were divided into 8 bins based on IRI fold changes, which were the average changes of *U2AF1*‐KD by two shRNAs. *, **, and *** denote *p* < 0.05, *p* < 0.01, *p* < 0.001, respectively, two‐tailed *t* test (except for panel D)

As some RBPs (such as HNRNPLL and PTBP1) were reported to regulate IR (Cho et al., 2014; Yap et al., 2012), we wondered whether dynamically changed expression of certain RBP(s) could also explain the regulated IR events during senescence. We thus screened for candidate RBPs with the following criteria: (1) significantly higher binding density of this RBP in retained introns compared to spliced introns in both in vitro senescence models (HFF and MRC‐5) and in vivo samples (dermal fibroblasts derived from young and aged healthy individuals) (Fleischer et al., 2018); (2) gene expression level is significantly different between earlier and later passaged cells in at least two out of six senescence models (HFF, MRC‐5, dermal fibroblasts, BJ, IMR90, and WI38) (Marthandan et al., 2015); and (3) gene expression level is larger than 10 RPKM (reads per kilobase million in RNA‐seq) in at least one of the HFF cell passages. We obtained 15 RBPs meeting all the above criteria, and the majority showed decreased expression during senescence (Figure S11). If downregulation of a candidate RBP regulates senescence‐related IR events, senescence‐associated phenotypes would be expected upon knockdown (KD) of such RBP. We thus focused on RBPs reported associating with senescence‐associated phenotypes, and selected out three candidate RBPs (SRSF1 (Blanco & Bernabeu, 2012; Fregoso et al., 2013), U2AF1 (Graubert et al., 2012; Shirai et al., 2015) and U2AF2 (McReynolds et al., 2012)). The classical senescence‐associated marker SA‐β‐Gal was applied for evaluating the senescence‐causing effect of knocking down candidate RBPs, and the results showed that knockdown of the splicing factor U2AF1 could induce higher SA‐β‐Gal staining in multiple human cells (HFF, HUVEC, and A549) (Figure 2a,b; Figure S12). We therefore chose U2AF1 (a component of U2 snRNP) for further analysis. U2AF1 showed higher binding density in regulated introns (than in spliced introns) and decreased expression in senescent HFF and MRC‐5 cells (Figure 2c,d; Figure S13). Moreover, similar tendency of U2AF1‐binding density and expression trend was observed in dermal fibroblasts from young and aged individuals (Figure S14 and Figure S15). In addition, *U2AF1* showed downregulated expression in three more replicative senescence models (BJ, IMR90, and WI38) (Figure 2d). These data suggested that U2AF1 downregulation was prevalent both in vitro and in vivo. Knocking down *U2AF1* (*U2AF1*‐KD) in HFF cells led to slower cell growth rate (Figure 2e) and increased expression of *CDKN2B* (coding cyclin‐dependent kinase inhibitor p15) (Figure 2f). In addition, *U2AF1*‐KD cells also showed decreased expression of *MKI67* (cell proliferation marker gene), the cycle‐related genes: *CDK1* (cyclin‐dependent kinase 1) and *CDK4* (cyclin‐dependent kinase 4) (Figure 2f). These multiple lines of evidence above support the notion that downregulation of U2AF1 does induce cellular senescence in HFF.

To examine whether *U2AF1*‐KD could affect IR, polyA^+^ RNA‐seq profiling was performed on both *U2AF1*‐KD and control HFF primary cells. For better reliability, two different shRNAs (each with two replicates) were used for transcriptome profiling. Strikingly, *U2AF1*‐KD with either shRNA induced hundreds of IR changes in HFF cells (see Table S2 for a complete summary of IRI values) and the IRI‐increased introns greatly outnumbered IRI‐decreased ones (Figure 2g). To explore whether *U2AF1*‐KD‐induced IR changes affect steady‐state expression, we grouped genes into four classes (up_IR, down_IR, stable_IR, and no_IR) as narrated above. Similarly, up_IR genes had decreased expression trend and down_IR genes had increased expression trend (Figure S16), while stable_IR and no_IR groups showed overall unchanged expression distribution. Moreover, the IRI increase degree linearly correlated with the extent of decreased gene expression when comparing *U2AF1*‐KD to control HFF cells (Figure 2h, Figure S17), indicating that *U2AF1*‐KD‐induced IR affected steady‐state gene expression of corresponding genes. All these results above demonstrate that downregulation of U2AF1 leads to prevalent IR and cellular senescence.

### 2.5 U2AF1‐mediated intron retention of *CPNE1* contributes to cellular senescence

**FIGURE 3:**
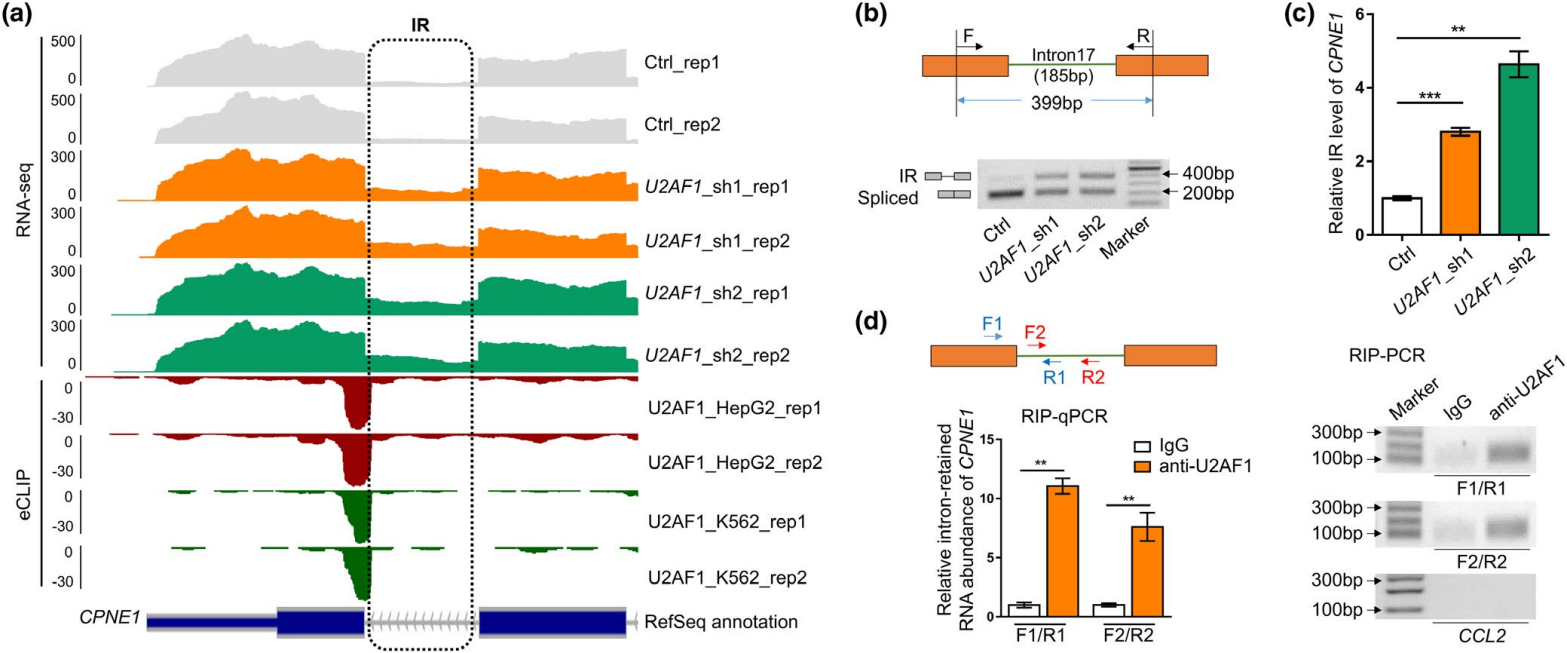
U2AF1 binds to intron border of *CPNE1* and leads to changed IR of *CPNE1*. (a) Wiggle plots showing intron retention supported by polyA^+^ RNA‐seq and U2AF1 binding supported by eCLIP reads near the last intron of *CPNE1*. (b) Changes of IR (upper bands) and spliced isoforms (lower bands) of *CPNE1* between *U2AF1*‐KD and control (Ctrl) cells detected by RT‐PCR. Primer design and size of two amplicons (399 bp for IR transcripts and 214 bp for spliced transcripts, respectively) were illustrated in the upper panel. (c) Relative intron retention ratio of *CPNE1* in *U2AF1*‐KD cells detected by qRT‐PCR. (d) U2AF1 binding to the retained intron of *CPNE1* detected by RIP‐qPCR and RIP‐PCR in HFF cells. Primers (F1‐R1; F2‐R2) designed to detect IR transcripts were illustrated in the left upper panel. IgG served as a negative control to U2AF1 antibody (anti‐U2AF1). *CCL2*, which showed no binding by U2AF1 in POSTAR2, served as a non‐specific control. *, **, and *** denote *p* < 0.05, *p* < 0.01, *p* < 0.001, respectively, two‐tailed *t* test with three replicates

**FIGURE 4:**
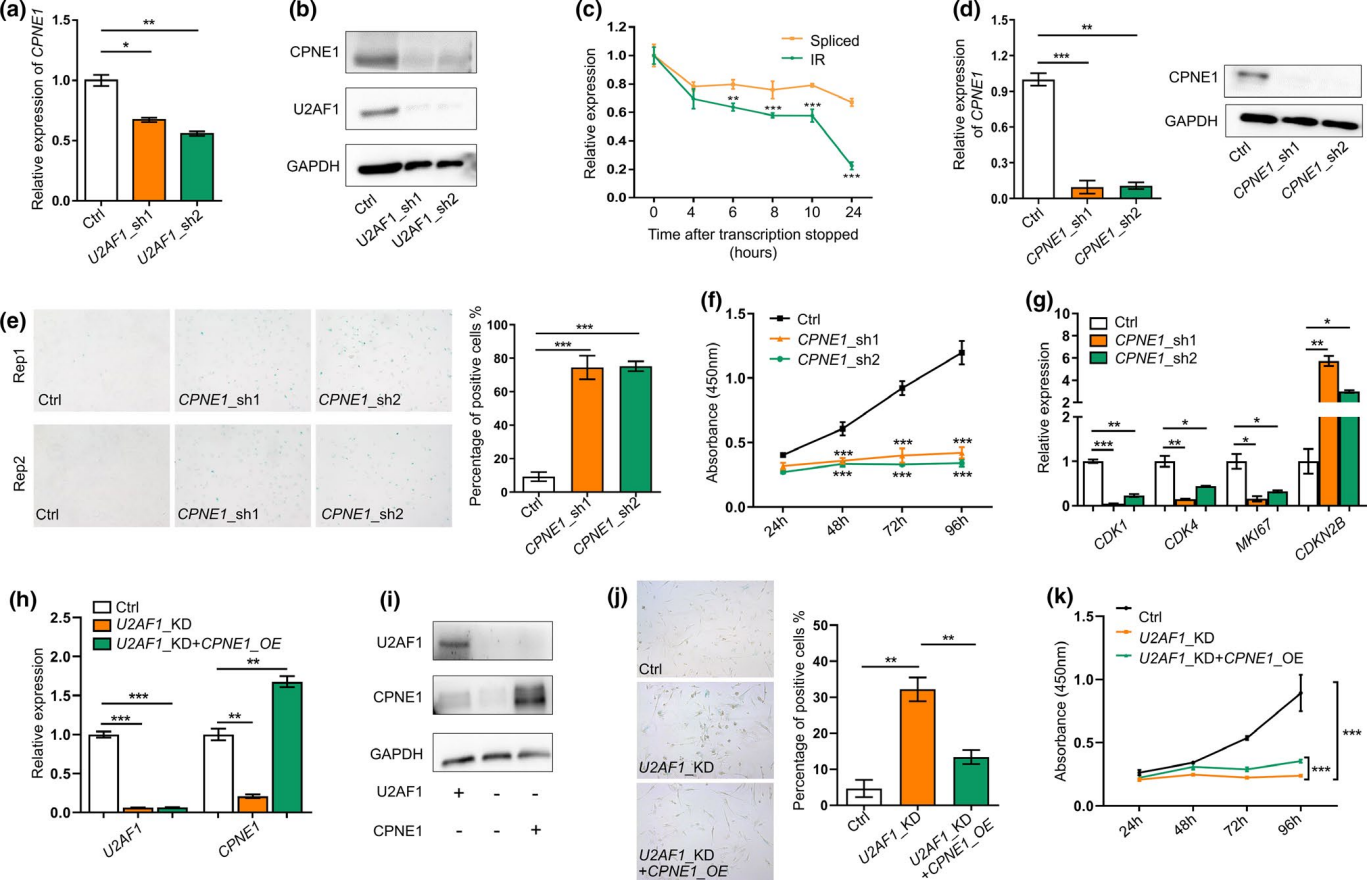
Intron‐retained *CPNE1 *has higher degradation rate and its downregulation contributes to senescence in HFF cells. (a) *CPNE1* gene expression in *U2AF1*‐KD cells detected by qRT‐PCR. (b) CPNE1 protein level in *U2AF1*‐KD HFF cells detected by Western blot. (c) Relative expression of IR and spliced transcripts of *CPNE1* detected by qRT‐PCR after inhibition of transcription with actinomycin D (10 µg/ml) treatment in HFF cells. (d) qRT‐PCR (left panel) and Western blot (right panel) validation of *CPNE1* knockdown by two shRNAs in HFF cells. (e) SA‐β‐Gal staining of control (Ctrl) and *CPNE1*‐KD HFF cells. Bar chart on right shows the percentage of SA‐β‐Gal‐positive staining cells. (f) Proliferation rate of *CPNE1*‐KD HFF cells measured by CCK‐8 assay. (g) Expression levels of *CDK1*, *CDK4*, *MKI67*, and *CDKN2B* detected by qPT‐PCR upon *CPNE1* knockdown. (h) *U2AF1* and *CPNE1* gene expression detected by qRT‐PCR before and after *CPNE1* overexpression (*CPNE1*‐OE) in *U2AF1*‐KD HFF cells. (i) Protein level of U2AF1 and CPNE1 detected by Western blot before and after *CPNE1*‐OE in *U2AF1*‐KD HFF cells. (j) SA‐β‐Gal staining of control (Ctrl), *U2AF1*‐KD, and rescue (*U2AF1*‐KD+*CPNE1*‐OE) HFF cells. Bar chart on right shows the percentage of SA‐β‐Gal‐positive staining cells. (k) Proliferation rate of Ctrl, *U2AF1*‐KD, and rescue HFF cells measured by CCK‐8 assay. *, **, and *** denote *p* < 0.05, *p* < 0.01, *p* < 0.001, respectively, two‐tailed *t* test with three replicates

To determine the causal role of U2AF1‐mediated IR to senescence, we screened for candidate genes that showed significant IR changes upon *U2AF1*‐KD with the following criteria: (1) It has altered IR both during *U2AF1*‐KD‐induced senescence and replicative senescence in HFF cells; (2) its intron retention level (IRI) was significantly changed upon *U2AF1*‐KD, and its expression was negatively correlated with IRI change; and (3) U2AF1 had binding peaks (supported by eCLIP data recorded in POSTAR2) in IRI‐changed introns or flanking exons. Six introns (in six genes) met all the above criteria (Table S3). As *CPNE1* is the gene with the most significant IRI change, we thus chose it for further intensive study. *CPNE1*, which encodes a calcium‐dependent membrane‐binding protein that activates AKT signaling cascade (Park et al., 2012), showed obvious IR upregulation of the last intron in *U2AF1*‐KD cells, and this retained intron can be bound by U2AF1 in two different cell lines (Figure 3a). Independent RT‐PCR (reverse transcription followed by polymerase chain reaction) and qRT‐PCR (quantitative RT‐PCR) confirmed increased intron retention level in *U2AF1*‐KD HFF cells (Figure 3b,c). Both RIP‐PCR (RNA immunoprecipitation followed by PCR) and RIP‐qPCR validated direct binding of U2AF1 to intron‐retained *CPNE1* transcript in HFF cells by two different PCR primer pairs (Figure 3d). In addition, *U2AF1*‐KD cells showed decreased expression of spliced *CPNE1* isoform (Figures 3, 4) and reduced CPNE1 protein level (Figure 4b). We next wondered how increased IR gave rise to reduced expression of *CPNE1*. RNA stability assay was performed to investigate the degradation rate between the IR and spliced transcripts, which showed that IR transcripts of *CPNE1* had a significantly higher degradation rate than the spliced ones (Figure 4c). This discovery suggests that U2AF1 knockdown causes increased intron retention, which competes with normally spliced isoform in the mRNA maturation process and degraded much faster, leading to the overall decreased RNA and protein level of *CPNE1*.

As downregulation of *U2AF1* resulted in elevated intron retention and decreased expression of *CPNE1* in HFF cells, we knocked down *CPNE1* to mimic the consequence of IR in *CPNE1* (Figure 4d) and then assayed senescence‐associated phenotypes to evaluate the contribution of IR in *CPNE1* to senescence. An increased SA‐β‐Gal staining and a significantly decreased cell proliferation rate were observed in *CPNE1*‐KD cells (Figure 4e,f). Moreover, *CPNE1*‐KD cells also showed increased expression of *CDKN2B*, and decreased expression of *CDK1*, *CDK4*, and *MKI67* (Figure 4g). Furthermore, we overexpressed *CPNE1* in *U2AF1*‐KD cells (Figure 4h,i) and observed that SA‐β‐Gal staining and cell proliferation rate were partially reversed (Figure 4j,k). These results suggest that *U2AF1*‐KD‐induced IR in *CPNE1* could contribute to cellular senescence.

To further confirm the prevalence of U2AF1‐CPNE1 regulation axis in senescence and aging, we analyzed public RNA‐seq data from in vivo aging model of dermal fibroblast from young to aged individuals and in vitro replicative senescence models (HFF, BJ, IMR90, and WI38). *U2AF1* showed a decreased expression trend from young to aged individuals (Figure S15), and the IRI level of *CPNE1* showed an increased tendency while the expression level exhibited a decreasing trend along dermal fibroblasts aging (Figure S18a). Similar changes of *U2AF1* and *CPNE1* (both IR and expression level) were detected in multiple replicative senescence models (Figure 2d, Figure S18b,c). These above results support that U2AF1‐mediated intron retention of *CPNE1* could be a common post‐transcriptional regulation mechanism in aging cells.

## 3 DISCUSSION

**FIGURE 5:**
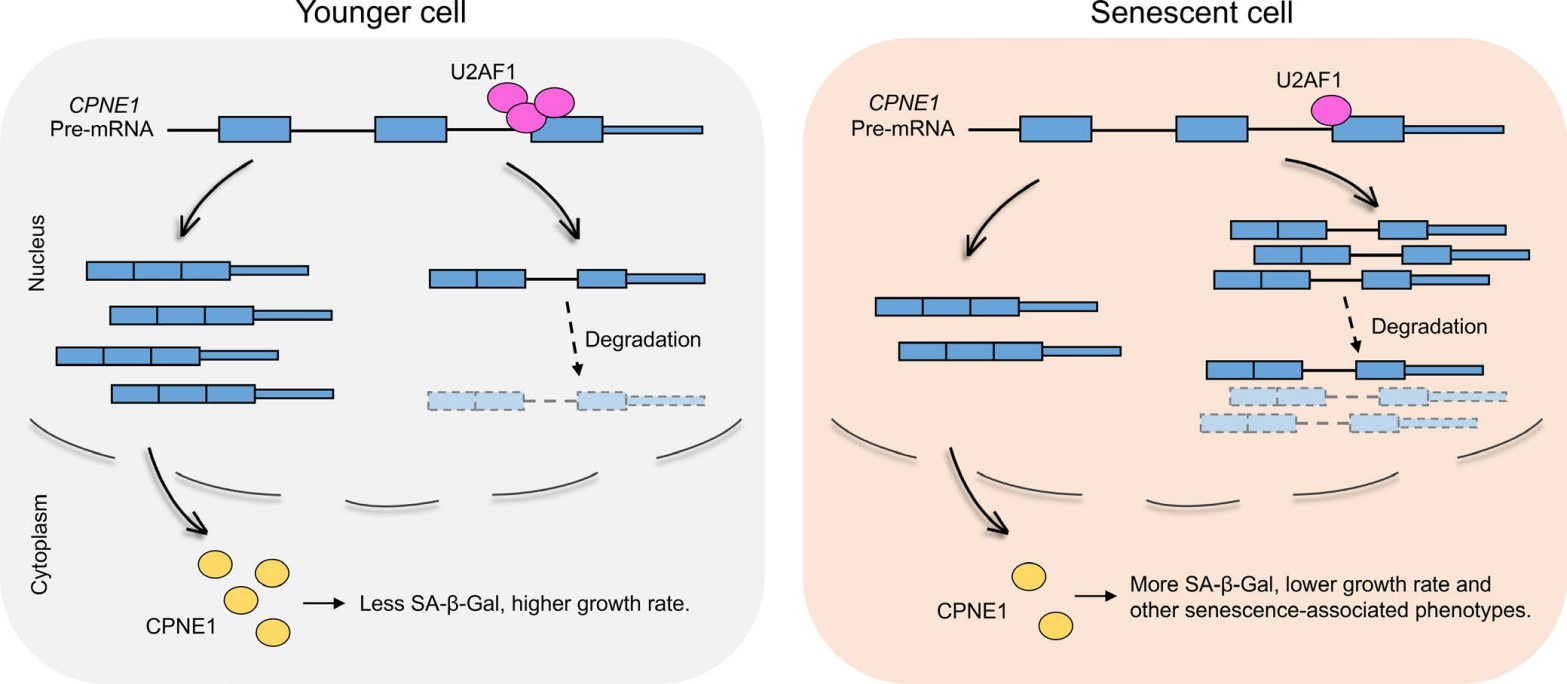
Working model for U2AF1‐mediated IR in regulating cellular senescence. Splicing factor U2AF1 binds to the last intron of *CPNE1* to promote efficient splicing in younger cells. Decreased expression of U2AF1 during senescence leads to increased intron retention of target gene such as *CPNE1*, which in turn generates less spliced transcripts. The intron‐retained transcripts compete with spliced ones and are rapidly degraded, resulting in the decreased normal translation template and reduced CPNE1 protein production, ultimately lead to senescence‐associated phenotypes

Although IR is the least well studied AS type in animals, recent progresses have demonstrated its underestimated role in diverse biological processes such as cell specification, development, and cancer (Braunschweig et al., 2014; Jung‐ et al., 2015; Pimentel et al., 2016; Smart et al., 2018; Wong et al., 2013). Cellular senescence has long been considered as an important cancer prevention mechanism, and it can be regulated at both transcriptional and post‐transcriptional levels (Kim et al., 2013; Serrano et al., 1997; Wei et al., 2015). Although previous studies had shown that the splicing factor SRSF1 can regulate IR of *ENG* gene during endothelial senescence (Blanco & Bernabeu, 2011), the causality of IR to senescence remains unclear. Recently, depletion of XAB2 was reported to induce intron retention of POLR2A and promote cell senescence (Hou et al., 2019). However, global analysis of IR in senescence models and cognition about the role of IR in senescence is still lacking. In this study, we showed that IR was prevalent in replicative human fibroblast models, wherein the intron retention levels dynamically changed and increased IR contributed to reduced gene expression. Analyzing dermal fibroblast dataset derived from young and old people (Fleischer et al., 2018) also indicated that prevalent intron retention is associated with fine‐tuned gene expression in individual aging (Figure S19). Such observation supports the notion that IR‐mediated gene regulation exists in cellular aging models both in vitro and in vivo. Of note, multiple genes with IR changes in our study that were either reported to regulate life span or age‐related functions (such as *SOCS2* (Casellas & Medrano, 2008) and *BAK1* (Someya et al., 2009)) or experimental validated to cause senescence in this study (such as *CPNE1*) were not presented in the HCSGD database (Dong et al., 2017), suggesting more senescence/aging‐associated genes with diverse regulation layers remain to be discovered. We further identified the splicing factor U2AF1 as one of the upstream factors to regulate global intron retention and senescence‐associated phenotypes. As a causality demonstration, *U2AF1*‐KD‐induced IR of *CPNE1* contributing to senescence was experimentally confirmed (Figure 5). Our study revealed a hidden layer of post‐transcriptional regulation of cellular senescence and may have implications in age‐related diseases including cancers.

By systematic analysis of polyA^+^ RNA‐sequencing data from replicative senescence models, we were able to reliably define intron retention events by calculating IRI based on our previously published method (Ni et al., 2016). Oligo(dT) enriched polyA^+^ RNA but not rRNA‐depletion RNA‐sequencing libraries were suitable for such IR analysis since rRNA‐depleted library contains incomplete or non‐polyadenylated mRNA that would cause false‐positive signals of IR. With the development of comparable computational approaches, it would be interesting to integrate all public available or homebrew polyA^+^ RNA‐seq data to explore not only the prevalence of IR in senescence but also cell type‐specific (such as fibroblast, endothelial cell, and stem cell) or senescence model‐specific (such as replicative senescence, stress‐induced premature senescence, and oncogene‐induced senescence) IR regulation in the future.

U2AF1 is a U2 snRNP auxiliary factor required for the binding of U2 snRNP to the pre‐mRNA branch site and 3ʹ‐splice‐site selection, which is essential for defining intron borders and subsequent splicing (Deschênes & Chabot, 2017). It had been clearly demonstrated that mutant U2AF1 could induce AS and alter expression of crucial genes and in turn lead to myelodysplastic syndromes (Graubert et al., 2012; Shirai et al., 2015). Furthermore, suppression of the major isoform U2AF1a increased un‐spliced transcripts of some cell‐cycle‐related genes and impaired mitosis in HeLa cells (Pacheco et al., 2006). These findings support that U2AF1 regulates cell viability or causes diseases mainly through impacting AS and expression level of relevant genes. Here, we discovered for the first time that U2AF1 deficiency could promote intron retention of hundreds of genes and the consequence of certain gene such as *CPNE1* could in turn lead to senescence‐associated phenotypes. Although U2AF1 is a core splicing factor required for global splicing, we found that U2AF1 knockdown caused IR changes in hundreds of introns but not all introns (Figure 2g). One possible explanation is that those IRI‐changed introns are more sensitive to the concentration of U2AF1. Multiple senescence models showed decreased expression of U2AF1 (Figure 2d), and one would expect that only introns sensitive to U2AF1 concentration may exhibit IR changes during senescence.

It is worth noting that U2AF1 is probably not the only regulator for IR in senescent HFF cells. We found that many annotated RBPs in POSTAR2 database, especially splicing factors, also showed binding potential to regulated introns (Figure S20). More RBPs showing decreased gene expression during senescence are worthy of systematic screening in the future. Based on the information above, we speculated that *U2AF1*‐KD‐induced IR would cover only partial of the IR events in replicative HFF senescence. In supporting this, there were 112 overlapping genes which have changed IR in both replicative senescence and *U2AF1*‐KD‐induced senescence in HFF cells (Figure S21), suggesting 16.4% (112/[573 + 112]) of IR‐changed genes during HFF replicative senescence could be potentially explained by reduced expression of *U2AF1*. In addition, genes in IR_up (384 genes) group greatly outnumbered those in IR_down (45 genes) group in *U2AF1*‐KD‐induced senescent cells, while the number of genes is relatively comparable between IR_up (368) and IR_down (333) group in replicative HFF. Noteworthy, as a splicing factor, U2AF1 also regulates other splicing events. Using rMATS (Shen et al., 2014) to analyze AS events between *U2AF1*‐KD and control HFF cells, we actually discovered other types of AS (including 1,275 exon skipping events, 41 alternative 5′ splice sites, 145 alternative 3′ splice sites, and 298 mutually exclusive exons). The numbers of total and senescence‐associated genes with splicing events for each AS type are shown in Table S4. Whether these AS events also contribute to *U2AF1*‐KD‐induced senescence deserves future study. Since senescence is a cancer prevention mechanism, we also explored the expression changes of *U2AF1* in diverse cancer types. We found five out of 33 cancer types showed significant *U2AF1* expression change between tumor and matched normal tissues (Figure S22). Four out of these five cancer types showed higher expression (colored red) of *U2AF1* in tumors while breast cancer (colored green) showed downregulated expression (Figure S22). As *U2AF1* showed heterogeneous expression profile among various cancer types, such heterogeneity deserves further study.

By combining RNA‐seq and public eCLIP data, we discovered 542 IR changes upon *U2AF1* knockdown (Figure S23) and 54 of these introns can be directly bound by U2AF1 in HFF cells (Figure S24). Similar binding ratio was also confirmed by simultaneously analyzing related data derived from other two cells (HepG2 and K562) (Figure S24), which had both polyA^+^ RNA‐seq and eCLIP data at ENCODE portal (see “Availability of data and materials” section). These results indicated that while U2AF1 could directly bind to and regulate some of the intron retentions, it might also indirectly interact with target RNAs (possibly mediated by spliceosome or other molecules) to influence the rest IR events, which were worth of further investigation. By candidate gene approach, we screened out *CPNE1* and provided evidence supporting that U2AF1 could directly bind near the 3′ splice site of *CPNE1*’s last intron to promote splicing. *U2AF1* deficiency led to increased intron retention and reduced expression of *CPNE1*, which ultimately resulted in senescence‐associated phenotypes. Additional candidate intron retentions that may contribute to senescence warrant further investigation.

The detailed mechanism of how decreased *CPNE1* expression leads to senescence also deserves additional study. CPNE1 is a calcium‐dependent phospholipid‐binding protein that plays a role in calcium‐mediated intracellular processes (Tomsig et al., 2004). CPNE1 can induce neurite outgrowth via an AKT‐dependent signaling cascade (Park et al., 2012, 2014). It also involves in TNF‐alpha‐induced NF‐kappa‐B transcriptional repression (Ramsey et al., 2008). Although the causal role of CPNE1 has been reported in multiple cancer types (Jiang et al., 2018; Liu et al., 2018; Tang et al., 2018), the function of CPNE1 in cellular senescence keeps unknown. We discovered for the first time that downregulation of CPNE1 could induce senescence‐associated phenotypes. Though the downstream signaling pathway awaits to be elucidated, it indicates a novel target for potential cancer treatment by inducing senescence, as promoting cancer cells to enter into senescence state has been proved to benefit cancer immunotherapy in a NK cell‐dependent manner (Ruscetti et al., 2018).

As intron‐retained transcripts could be either detained in nucleus and degraded by exosome or transported to cytoplasm and eventually degraded by NMD pathway (Wong et al., 2016), we examined which cellular component the intron‐retained *CPNE1* transcript tend to locate. By separating nuclear and cytoplasmic fractions of HFF cells, we found IR signal of *CPNE1* specifically located in nucleus (Figure S25). Some reports have already proved that exosome could degrade nuclear intron‐retained transcripts. For example, exosome subunit Rrp6 in fission yeast was involved in degradation of nuclear intron‐retaining transcripts to regulate gene expression (Lemieux et al., 2011). PABPN1 auto‐regulated its expression level by promoting retention of the 3′‐terminal intron, which was then degraded by nuclear exosome (Bergeron et al., 2015). These results imply that IR transcripts of *CPNE1* might be prevented from transporting to cytoplasm and degraded in nucleus, as supported by the observation that intron‐retained transcripts degraded faster than spliced ones (Figure 4c).

## 4 EXPERIMENTAL PROCEDURES

### 4.1 Datasets

PolyA^+^ RNA‐seq data (available on Gene Expression Omnibus [GEO] under accession number GSE63577) are derived from five human senescent cell models: five population doubling (PD) time points for both HFF and MRC‐5 cells; two PD time points for BJ, WI‐38, and IMR‐90 cells (Marthandan et al., 2015). The data contain three biological replicates for each PD. The RNA‐seq dataset derived from human dermal fibroblasts of different ages of individuals is available on GEO under accession number GSE113957 (Fleischer et al., 2018). U2AF1 eCLIP wiggle files for RBP‐binding sites analysis were downloaded from The Encyclopedia of DNA Elements (ENCODE) (https://www.encodeproject.org/) (ENCODE Project Consortium, 2012) with the following identifiers: ENCFF553KUR, ENCFF901KXE, ENCFF672OHA, and ENCFF822MGE. PolyA^+^ shRNA RNA‐seq data of U2AF1 knockdown are available on ENCODE portal under experiment ID ENCSR372UWV and ENCSR342EDG for cell lines HepG2 and K562, respectively. eCLIP data of U2AF1 knockdown are available on ENCODE portal under experiment ID ENCSR328LLU and ENCSR862QCH for cell lines HepG2 and K562, respectively.

### 4.2 Process of RNA‐seq data

RNA‐seq reads were mapped to human genome hg19 using STAR (version 2.5.3a) (Dobin et al., 2013), and the uniquely mapped reads were kept for further analysis. Gene body coverage analysis was performed using *geneBody_coverage* module from the RSeQC python package (Wang et al., 2012). Gene expression level for each sample specified as FPKM (fragments per kilobase of transcript per million fragments mapped) was calculated by StringTie (Pertea et al., 2015). As genes with low expression levels are more susceptible to statistical errors regarding IR, we only include genes with FPKM >1 in at least one group for downstream analysis. Gene expression changes between two conditions were estimated using DESeq2 R package (Love et al., 2014). rMATS (v 4.0.2) (Shen et al., 2014) was used to analyze differential AS events between *U2AF1*‐KD and control HFF cells, with inclusion level difference >10% and FDR <0.05.

### 4.3 Calculation of intron retention index (IRI)

Intron information was extracted from the RefSeq gene annotation file downloaded from the UCSC Genome Browser database (Tyner et al., 2016). To reliably estimate the IR levels, it is crucial to minimize potential interference coming from alternative exons/introns of multiple isoforms. For this purpose, only the shared intronic (exonic) regions in all annotated isoforms were considered as intronic (exonic) regions. IR events were determined using our established intron retention index (IRI) algorithm, which defined IRI as the ratio of read density of the intronic region and that of corresponding flanking exonic regions (Ni et al., 2016). To reduce false positive, introns with read coverage regions less than 50% of the intron length were removed. Introns with an IRI value greater than 1 in any sample were discarded to minimize the negative impact due to missing annotations, so the remained retained introns all have an IRI value ranging from 0 to 1. To reliably call an intron retention event, we also applied a retention cutoff of 10% (i.e., IRI ≥ 0.1 as the threshold to defined a trustable retained intron), which was used for a previous study (Schmitz et al., 2017). In‐house scripts for IRI calculation are available at https://github.com/gouge611/intron_retention_analysis_senescence.

### 4.4 Classification of IR events

For replicative cellular senescence models (HFF and MRC‐5) with five time points, each intron was classified into five groups according to the following criteria:

- Intron with IRI <0.1 across all time points (i.e. *max*(*X*) <0.1) were defined as constitutively spliced introns (no_IR);
- Intron with *max*(*X*)−*min*(*X*) <0.1 were defined as no significant change in IRI (stable_IR);
- Intron with *max*(*X*)−*min*(*X*) ≥0.1 and *Cor*(*X*, *L*)>0.7 were defined as continuous up (up_IR);
- Intron with *max*(*X*)−*min*(*X*) ≥0.1 and *Cor*(*X*, *L*)< −0.7 were defined as continuous down (down_IR);
- The rest were discarded for downstream analysis due to irregular change pattern.
*X* = (*X*_1_, *X*_2_, …, *X*_n_) is a vector containing IRI values of all time points of the intron. *max*(*X*) and *min*(*X*) are the maximum and minimum *X* value (IRI) across all time points, respectively. *L* = (*L*_1_, *L*_2_, …, *L*_n_) is a monotonically increasing sequence, which was designed to simulate entirely increasing trend of time‐scale data. In this case, the length of vector *L* consists with the length of *X*, the initial value *L*_1_ is 0, the last value *L_n_* is 1, and the difference between the consecutive values (i.e. *L_n_−L_n−1_*) is $\frac{1}{n‐1}$. *Cor*(*X,L*) is the Pearson correlation coefficient between *X* and *L*.
  The goal of the first criterion was to firstly classify events with IRI value lower than retention cutoff all over time points. After filtering out those constitutively spliced introns, the goal of the second criterion was to find IR events with little change in IRI value across all time points. The goal of the third and fourth criteria was to determine continuously increase or decrease IR events with change greater than retention cutoff and continuous change trend. If tendency of IR is continuously change during senescence, the absolute value of correlation coefficient between time‐scale IRI values *X* and monotonically increasing sequence *L* would be expected to higher than those with irregular change pattern. Plus or minus of correlation coefficient indicates increasing or decreasing trend.

For polyA^+^ RNA‐seq data with only two conditions, the criteria are similar but slight different because of distinct data type:

- Intron with IRI <0.1 in both two conditions was defined as constitutively spliced introns (no_IR);
- Intron with $\left|\Delta IRI\right|<0.1$ was defined as no significant change in IRI (stable_IR);
- Intron with *∆IRI* ≥0.1 and the *p*‐value of two‐sided *t* test less than 0.05 was defined as continuous up (up_IR);
- Intron with *∆IRI *≤ −0.1 and the *p*‐value of two‐sided *t* test <0.05 were defined as continuous down (down_IR);
- The rest were discarded for downstream analysis due to irregular change pattern.

### 4.5 Analysis of splice‐site strength

The maximum entropy model (Yeo & Burge, 2004) was used to estimate the strength of donor splice sites and acceptor splice sites of each intron.

### 4.6 RBP‐binding site analysis

Genomic coordinates of RBP‐binding sites based on experimental evidences (HITS‐CLIP, CLIP‐seq, PAR‐CLIP, iCLIP, and eCLIP) were downloaded from the POSTAR2 database (Hu et al., 2017). As the human reference genome version used in POSTAR2 is hg38, liftOver (Tyner et al., 2016) was used to transform the genomic coordinates from hg38 to hg19 with default parameters.

### 4.7 Cell culture and lentiviral transfection

Primary HFF cells were kindly provided by Stem Cell Bank, Chinese Academy of Sciences. Cells were cultured in DMEM (Gibco) supplemented with 15% FBS (Gibco) at 37°C in a humidified, 5% CO_2_ incubator. For A549, HUVEC, and 293 T, cells were cultured in 10% FBS of DMEM. For stable knockdown of target genes, we applied lentivirus transfection‐mediated gene‐silencing strategy. shRNAs (shRNA sequences are listed in Table S5) were annealed and then cloned into pLKO.1 vector. 293 T cells grown in 6‐well plates were transfected with 1 μg constructed vectors or negative control vectors pLKO.1 with VSVG and gag/pol encoding plasmids using Lipofectamine 2000 (Invitrogen). After culturing for 24 h, the virus supernatant was harvested to infect HFFs in six‐well plates seeded one day ahead. After incubation over 24 h, HFFs were screened by 2.5 μg/ml puromycin for 1 day. The surviving cells were cultured for two more days and then used for cell proliferation assay and RNA extraction.

### 4.8 RNA extraction, qRT‐PCR, and Western blot

Total RNA and protein were isolated with TRIzol reagent according to manufacturer's instructions (Invitrogen). Then, polyA^+^ RNA was reversely transcribed into cDNA with oligo‐(dT) primer (AAG CAG TGG TAT CAA CGC AGA GTA CTT TTT TTT TTT TTT TTT TTT TTT TTT TTT TVN) using FastQuant RT Kit (Tiangen). Gene expression at the RNA level was quantified by qRT‐PCR using 2× SYBR mix (Vazyme). *GAPDH* served as an internal control. Then, the reaction was run on Bio‐Rad CFX manager machine.

For Western blot, protein was incubated at 95°C for 10 min before performing SDS‐PAGE. The primary antibodies for U2AF1 (ab86305; 1: 2000, Abcam), CPNE1 (ab155675, 1: 1000, Abcam), and GAPDH (AB2000, 1: 5000, Abways) were incubated with membrane for 2 h at room temperature. The blots were then washed and incubated with second antibody (Goat Anti‐Rabbit, M21002L, 1: 5000, Abmart) for 1 h at room temperature. After washing in TBST, blots were exposed by Tanon Chemiluminescent Imaging System.

### 4.9 Cell proliferation assay

Cell proliferation assay was performed on cultured cells at four time points (24, 48, 72, and 96 h). Cells were counted and seeded in 96‐well plates with 1000 cells per well and four replicates for each time points. Cell Counting Kit‐8 (CCK‐8) reagent (Dojindo) was diluted with DMEM according to the manufacturer's protocol and then added to each testing wells. Then, cells were incubated at 37°C for another 2 h and then the absorbance of each well was measured at 450 nm by a microplate reader (Bio‐Rad).

### 4.10 SA‐β‐Gal staining

Senescence Cells Histochemical Staining Kit (CS0030‐1 KT; Sigma) was used for senescence‐associated‐β‐Gal activity detection. Cells were seeded in 12‐well plates at 70% confluence. Then removing the DMEM medium, cells were washed twice with 1× PBS and incubated for 6–7 min with 0.6 ml 1× fixation buffer per well. After fixation, cells were washed twice with 1× PBS. The staining solution mixture (10× staining solution, reagent B, reagent C, X‐gal solution and ddH_2_O, prepared according to the manufacturer's protocol) was added into cells. Cells were incubated at 37°C overnight and then observed under an inverted microscope (Olympus).

### 4.11 RNA‐seq library construction

We constructed the dUTP‐based strand‐specific RNA‐seq library by RNA HyperPrep Kit (KAPA). 500 ng purified total RNA of each sample was processed for poly(A) enrichment and fragmentation. Then, 1st‐strand cDNA and 2nd‐strand cDNA were synthesized from fragmented RNA, followed by A‐tailing and adapter ligation. Finally, adapter‐ligated library DNA was amplified by PCR. Library concentration was determined by Qubit dsDNA HS Assay Kit (Invitrogen) according to manufacturer's instructions. Then, the libraries were sequenced via Illumina HiSeq X Ten.

### 4.12 Validation of intron retention by RT‐PCR and qRT‐PCR

Cells with or without knockdown of *U2AF1* were collected for RNA extraction. The RNA isolation and cDNA synthesis were performed as mentioned above. For RT‐PCR, a pair of primers were designed as shown in Figure 3b. 100 ng cDNA was used for each PCR reaction, and PCR products were detected by agarose gel electrophoresis. For qRT‐PCR, the forward and reverse primers of *CPNE1* were designed at the retained intron and upstream/downstream exons for quantitating the intron retention. And the other pair of primers were designed at two adjacent exons for quantitating the RNA levels of candidate genes. Thus, the ratio of quantitative value of the former to the latter reflected the ratio of intron retention. All primer sequences are listed in Table S5.

### 4.13 RNA stability assay

HFF cells were seeded in a 12‐well plate in advance. Then, cells were treated with actinomycin‐D (10 µg/ml) for 0, 4, 6, 8, 10, and 24 h, respectively. Total RNAs were extracted with TRIzol reagent for each treatment. The IR and spliced transcripts of *CPNE1* were quantified by qRT‐PCR using primers specifically targeting the retained intron and exon junction, respectively.

### 4.14 RNA immunoprecipitation coupled with RT‐PCR and qRT‐PCR

Firstly, we added 40 µl protein G beads to a new RNase‐free tube and washed beads twice with 1 ml cold RIP lysis buffer (50 mM Tris‐HCl pH 7.4, 250 mM NaCl, 5 mM EDTA, 1% NP40, 0.5 mM DTT, 100 U/ml RNase inhibitor, 1× protease inhibitor) on a magnetic stand. Then, we resuspended beads with 100 µl cold RIP buffer and separated beads equally into two parts. Beads were incubated with anti‐U2AF1 antibody (ab86305, 4 µg, Abcam) and Rabbit IgG antibody (B30011S, 4 µg, Abmart) separately at 4°C for 4 h. Next, two 10‐cm dishes of HFF cells were prepared and washed once with cold PBS after aspirating the medium. 1 ml PBS was added and dishes were placed on ice, followed by irradiating cells under 200 mJ/cm^2^ at 254 nm using UV crosslinker. Then, cells were immediately washed twice with cold PBS. 1 ml cold RIP lysis buffer was added to each dish and cells were incubated on ice for 10 min. Then, cells were collected into two RNase‐free tubes by scraping with a cell lifter. Cells were sonicated for 5 × 30 s by Bioruptor and then centrifuged at 16,000 g, 4°C for 10 min to collect the supernatant. The two tubes of supernatant were then added into anti‐U2AF1‐beads and IgG‐beads separately and incubated at 4°C for 4 h. After that, the tubes were put on a magnetic stand for 1 min to remove the solution and beads were washed three times with cold RIP buffer. 20 µl proteinase K solution was added (100 mM Tris‐HCl pH 7.4, 50 mM NaCl, 10 mM EDTA, 4 mg/ml proteinase K) to the beads and beads were incubated at 37°C for 20 min to digest protein. The tubes were put on magnet once again to remove the solution, and Urea solution (100 mM Tris‐HCl pH 7.4, 50 mM NaCl, 10 mM EDTA, 7 M urea) was added and then incubated for 20 min at 37°C. Finally, RNA was extracted with TRIzol reagent and reversely transcribed into cDNA. For RIP‐qPCR, the RNA levels were quantified using 2×SYBR mix (Vazyme) and the reaction was run on Bio‐Rad CFX manager machine. For RIP‐PCR, 300 ng cDNA was used for each reaction. PCR products were detected by agarose gel electrophoresis. The primers designed for RIP‐qPCR and RIP‐PCRare shown in Figure 3d, and the sequence information is listed in Table S5.

### 4.15 Isolation of nuclear and cytoplasmic fractions

HFF cells were cultured in a 10‐cm dish until 90% confluence. Then, cells were trypsinized, washed twice with cold PBS, and resuspended in 500 µl cold lysis buffer (20 mM Tris‐HCl pH 7.4, 150 mM KCl, 100 mM NaF, 1 mM DTT, 1% NP40, 1.5 mM MgCl_2_, 100 U/ml RNase inhibitor, 1× protease inhibitor). Cell lysate was incubated on ice for 10 min and then centrifuged at 500 g for 5 min at 4°C. Then, the supernatant was carefully pipetted into a new tube. The pellet was washed once with 200 µl cold lysis buffer and centrifuged again at 500 g for 5 min. The supernatant was moved into the tube once again and mixed with the supernatant collected from the last step to make the cytoplasmic fraction, and the pellet left was the nuclear fraction. Finally, RNA was extracted with TRIzol reagent and reversely transcribed into cDNA, which was used for quantification of interested genes/transcripts.

## CONFLICT OF INTERESTS

None declared.

## AUTHOR CONTRIBUTIONS

TN and GW conceived the overall project and supervised its execution. JY performed statistical and bioinformatics analyses. HZ supervised the statistical analysis. HF participated in the design of the project. DD, XL, and TS designed and performed the experiments. JY, DD, GW, and TN wrote and revised the manuscript. All authors reviewed the results and approved the manuscript.

## Supporting information

Fig S1‐S25

Table S1

Table S2

Table S3

Table S4

Table S5

## Data Availability

RNA‐seq raw data with and without U2AF1 knockdown in HFF cells can be found at NCBI Sequence Read Archive (SRA) under accession number PRJNA565612.
