# Supplementary material for: Prevalent intron retention fine‐tunes gene expression and contributes to cellular senescence
Source: Aging Cell. 2020 Dec 4;19(12):e13276. doi: 10.1111/acel.13276 (PMC7744961; doi:10.1111/acel.13276)
Supplement: Supplementary file 1 — Fig S1‐S25 [file ACEL-19-e13276-s001.docx]

Prevalent intron retention fine-tunes gene expression and contributes to cellular senescence

Jun Yao^1,†^, Dong Ding^1,†^, Xueping Li^1^, Ting Shen^1^, Haihui Fu^1^, Hua Zhong^2^, Gang Wei^1,*^, Ting Ni^1,*^

^1^State Key Laboratory of Genetic Engineering, Collaborative Innovation Center of Genetics and Development, Human Phenome Institute, School of Life Sciences, Fudan University, Shanghai 200438, P.R. China

^2^Department of Population Health, NYU Langone School of Medicine, 180 Madison Avenue, 4th Floor, Room 452, New York, NY 10016, USA

^†^ Contributed equally

^*^ Correspondence should be addressed to G.W. (gwei@fudan.edu.cn) and T.N. (email: tingni@fudan.edu.cn).

#####

## Supplemental Figures


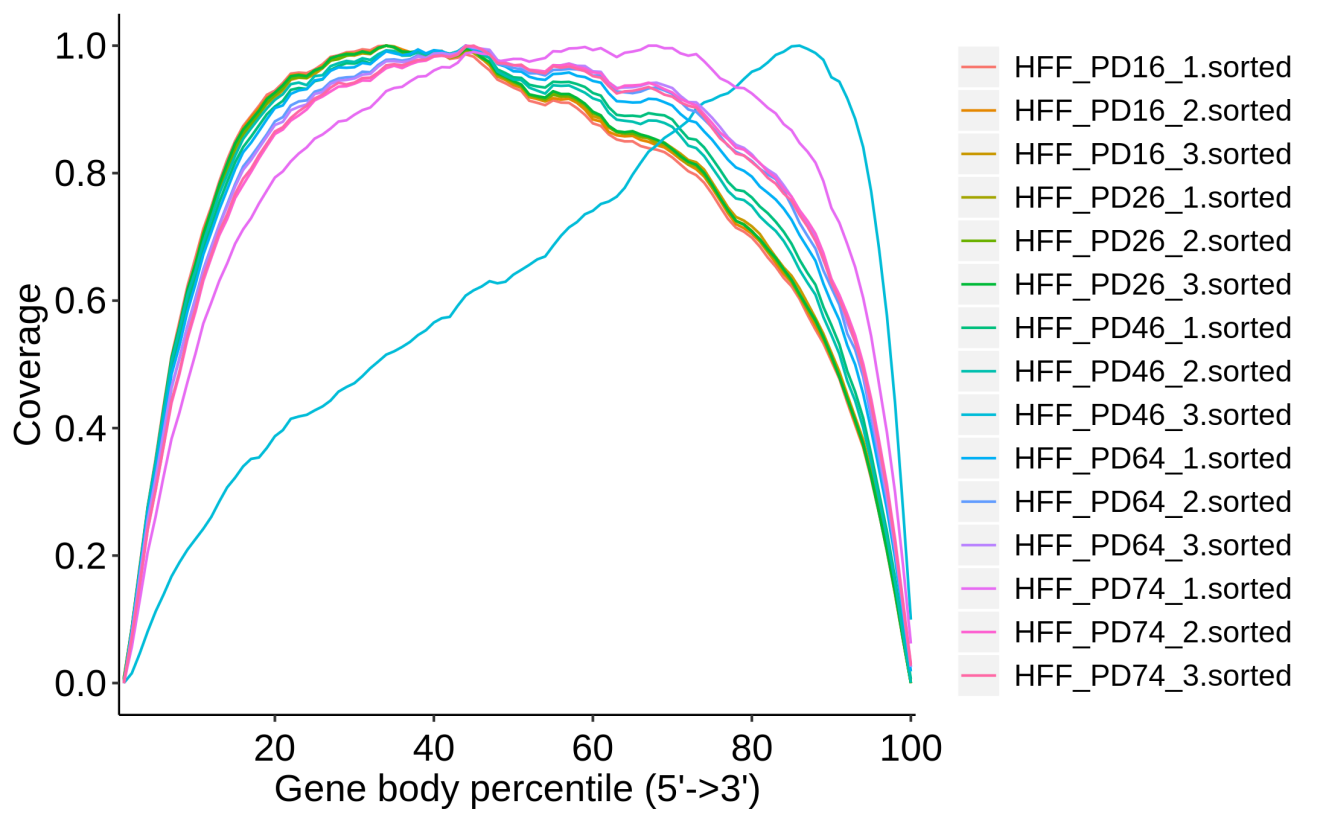


Figure S1: Coverage plot of mapped RNA-seq reads along the gene body. All transcripts were scaled to 100 nt and the read coverage was normalized to each nucleotide position. Sample PD46_3 has obvious 3′-end bias. To reduce false positive signals derived from such a bias, we removed this sample from analyses throughout the manuscript.


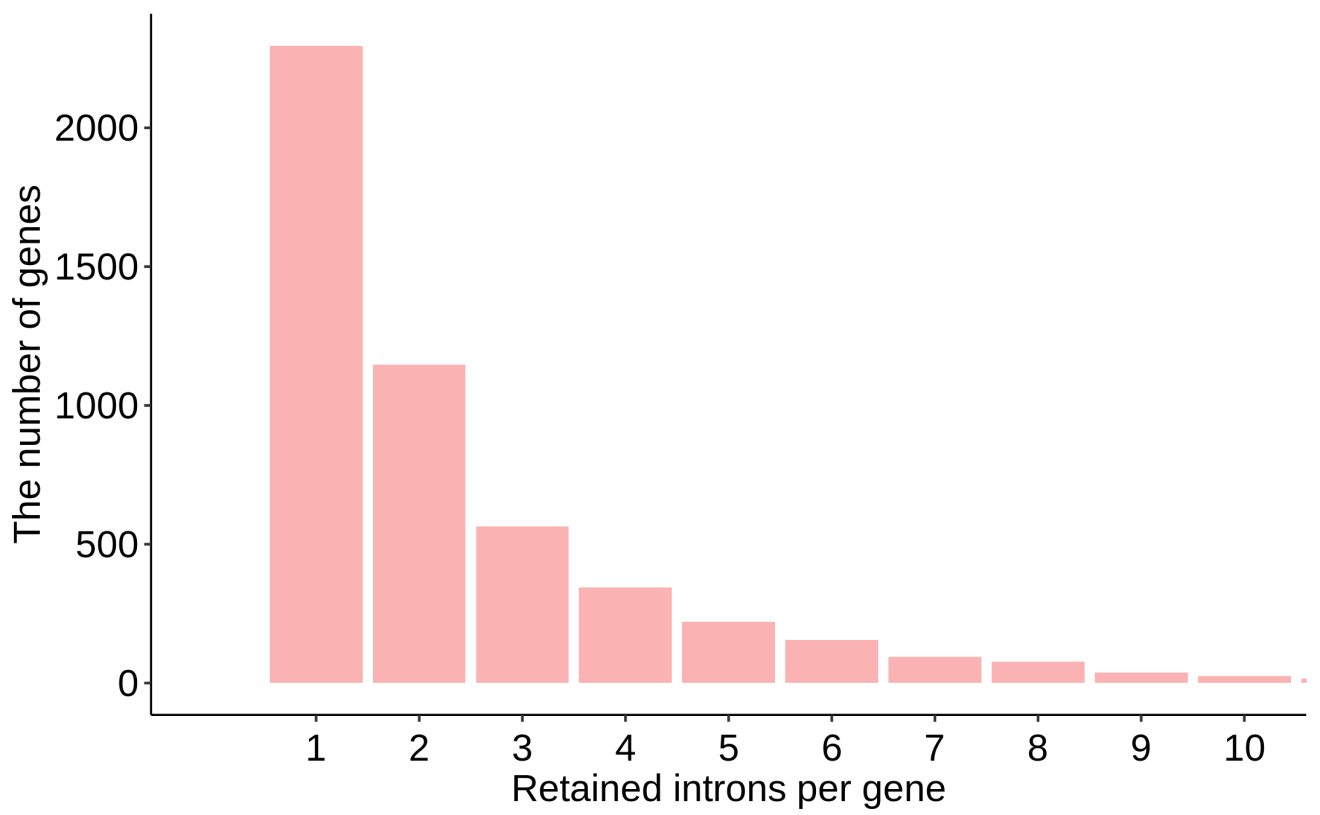


Figure S2: Histogram of the number of genes with different number of retained introns in HFF cellular senescence.

#####


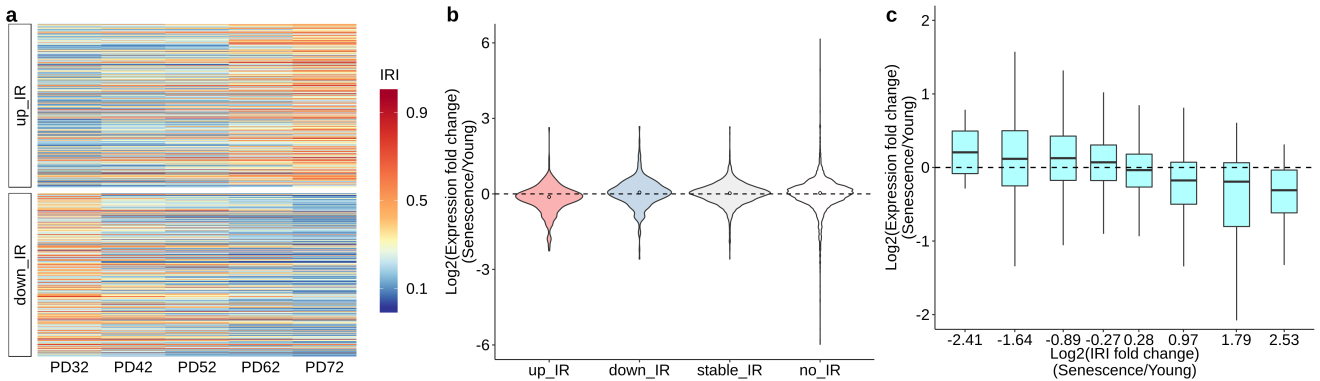


Figure S3: **IR changes negatively correlated with gene expression during MRC-5 cellular senescence.** (a) Heatmap showing IRI continuously up- and down-regulated (up_IR and down_IR). (b) Violin plot showing the distribution of gene expression changes between senescent (PD72) and young MRC-5 cells (PD32) for genes with different IR changes. (c) Box plot showing the negative correlation between IR changes and corresponding gene expression alteration. Genes were divided into 8 bins based on IRI fold changes between senescent (PD72) and young (PD32) MRC-5 cells.

#####


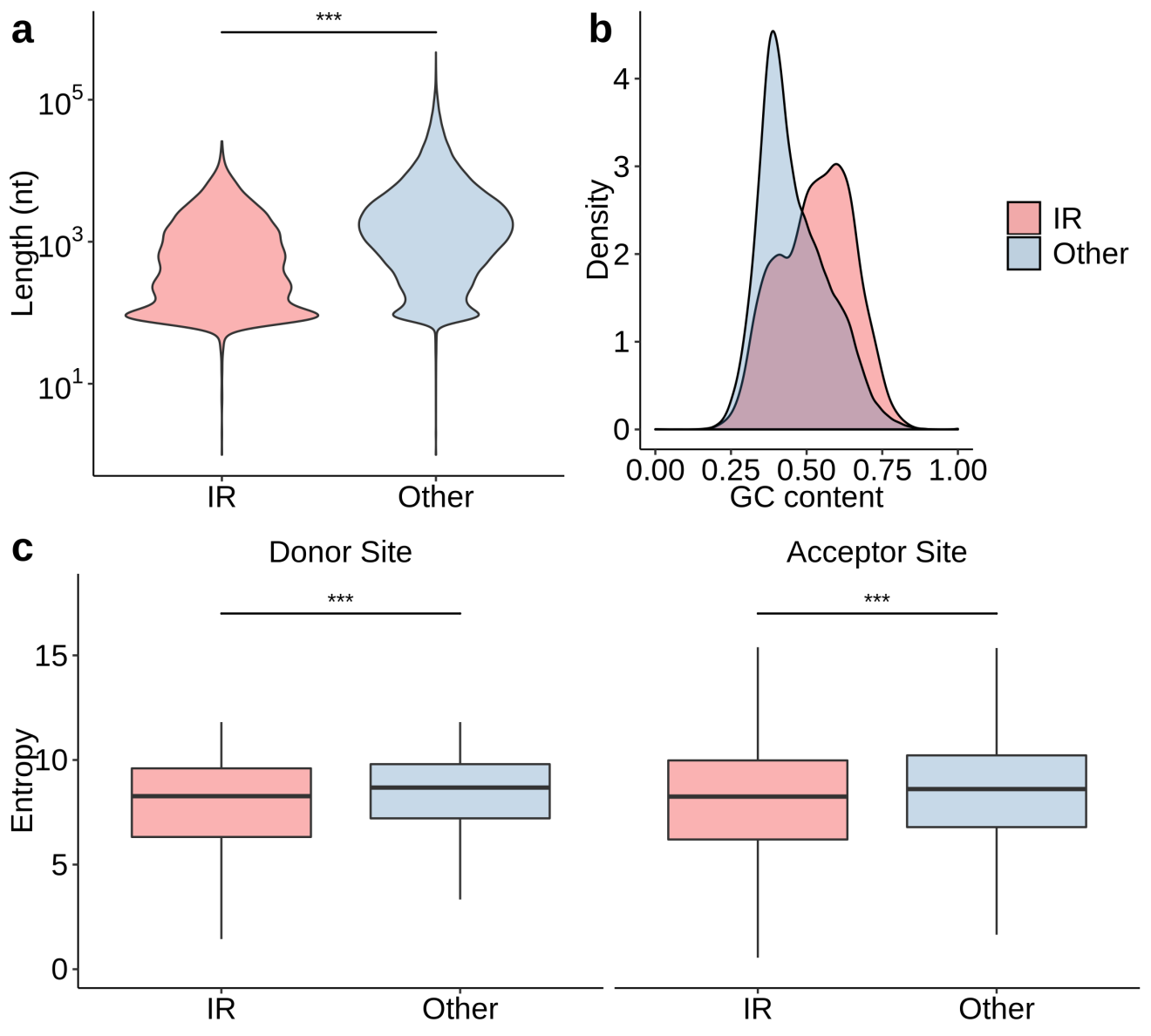


Figure S4: **Characteristics of retained introns in HFF cellular senescence.** (a) Violin plot showing the log_10_ transformed length distribution of retained (IR) and non-retained (Other) introns. (b) Density plot of the GC content of retained and non-retained introns. (c) Splicing strength (measured by maximum entropy) distribution of donor and acceptor splice sites of retained and non-retained introns. *** indicates $P<0.001$, two-sided Wilcoxon test.

#####


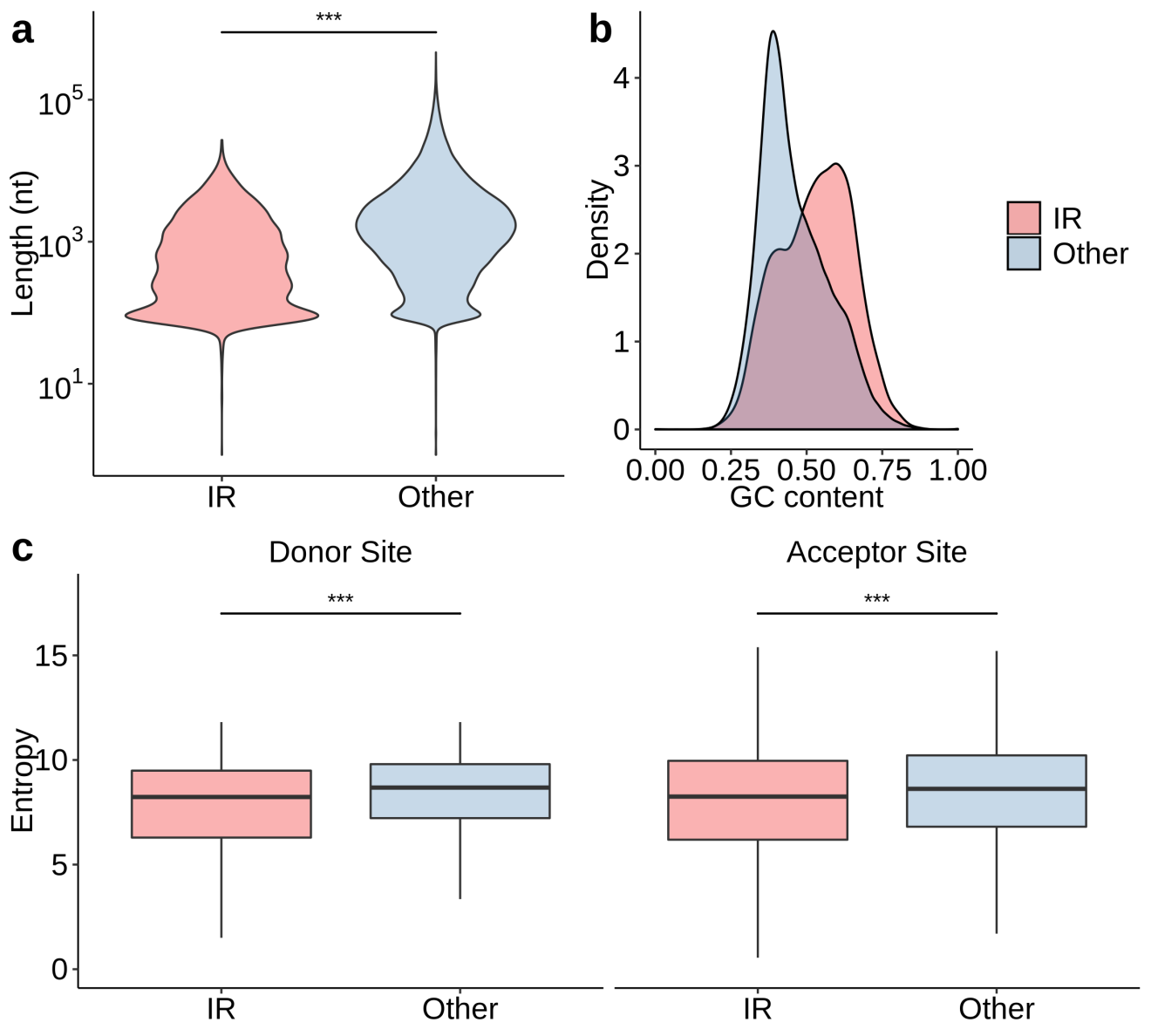


Figure S5: **Characteristics of retained introns in MRC-5 cellular senescence.** (a) Violin plot showing the distribution of length of retained (IR) and non-retained (Other) introns. (b) Density plot of the GC content of retained and non-retained introns. (c) Comparison of splicing strength (measured using maximum entropy) of donor and acceptor splicing site for retained and non-retained introns. *** indicates $P<0.001$, two-sided Wilcoxon test.

#####


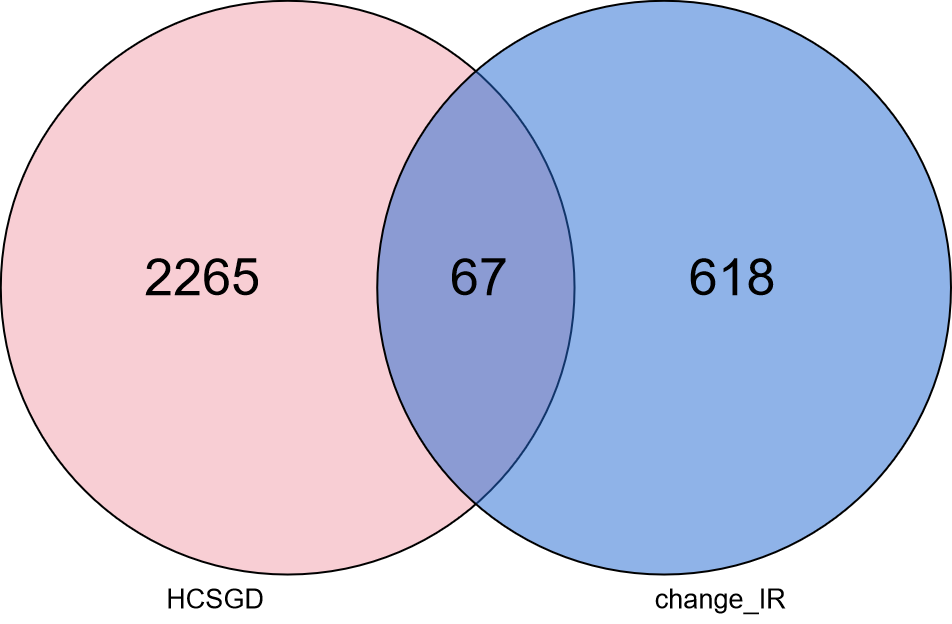


Figure S6: Venn diagram showing relation between genes with regulated IR (658 genes in total) and senescence-associated genes archived in HCSGD (http://bioinfo.au.tsinghua.edu.cn/member/xwwang/HCSGD).

#####


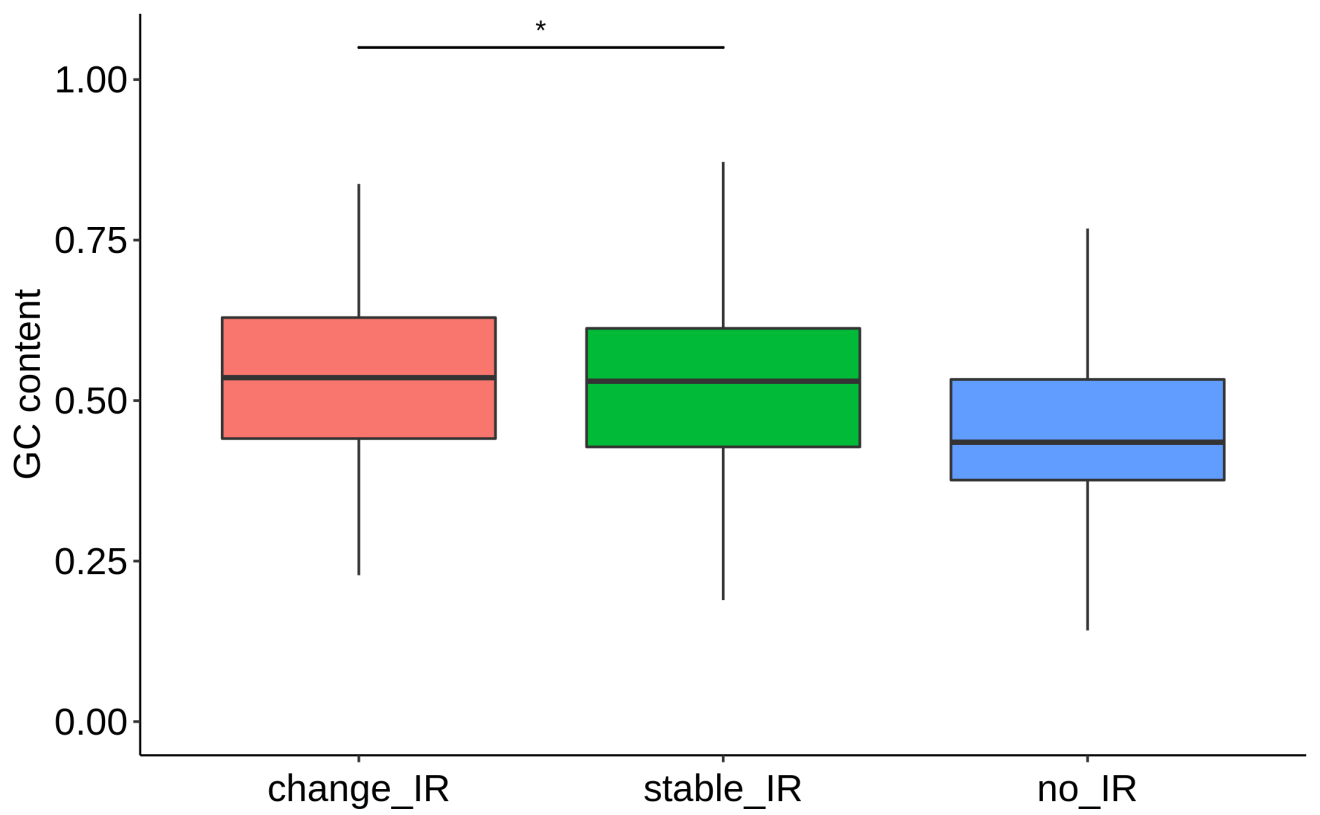


Figure S7: Boxplot showing the GC content distribution of regulated (change_IR) and constitutively retained (stable_IR) introns. * indicates $P<0.05$, one-sided Wilcoxon test.

#####


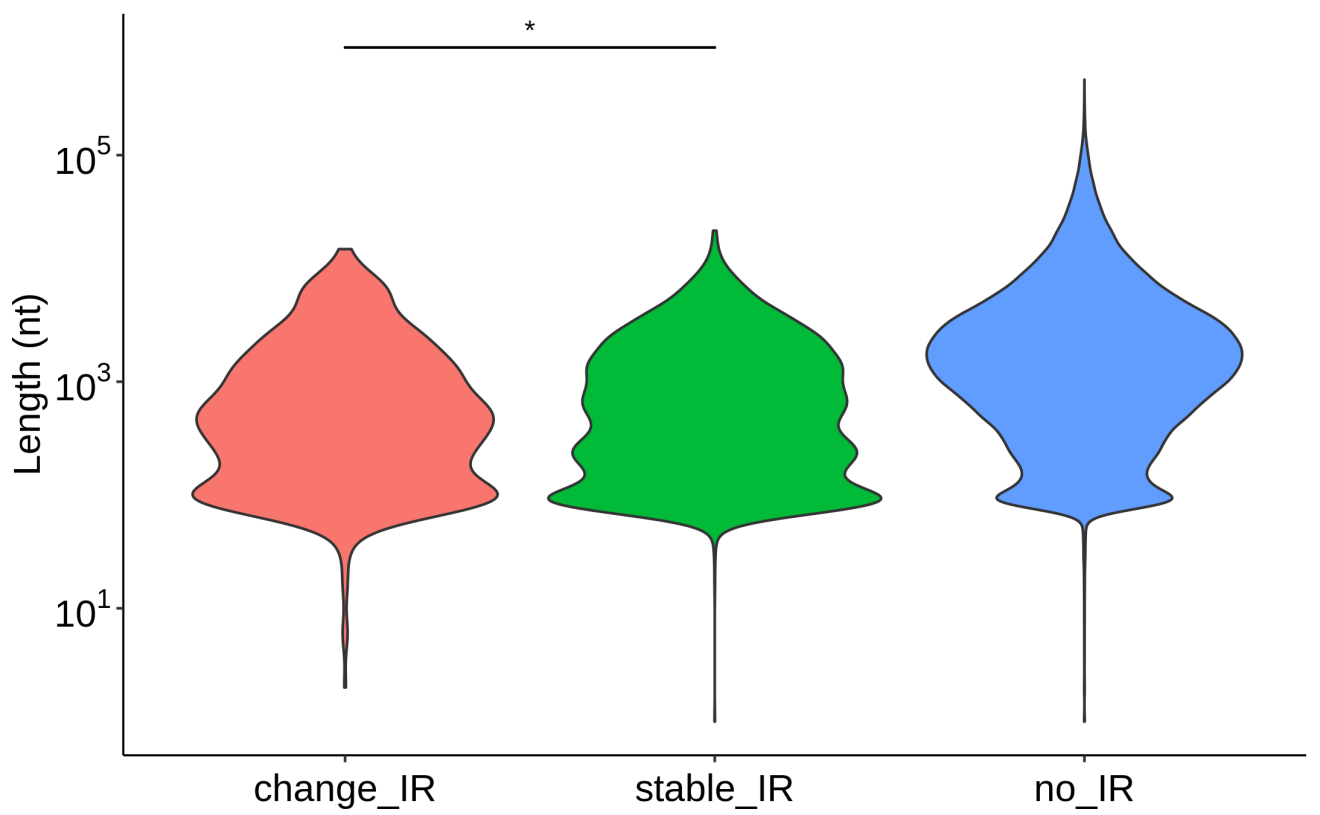


Figure S8: Violin plot showing the distribution of length for regulated (change_IR) and constitutively retained (stable_IR) introns. * indicates $P<0.05$, one-sided Wilcoxon test.

#####


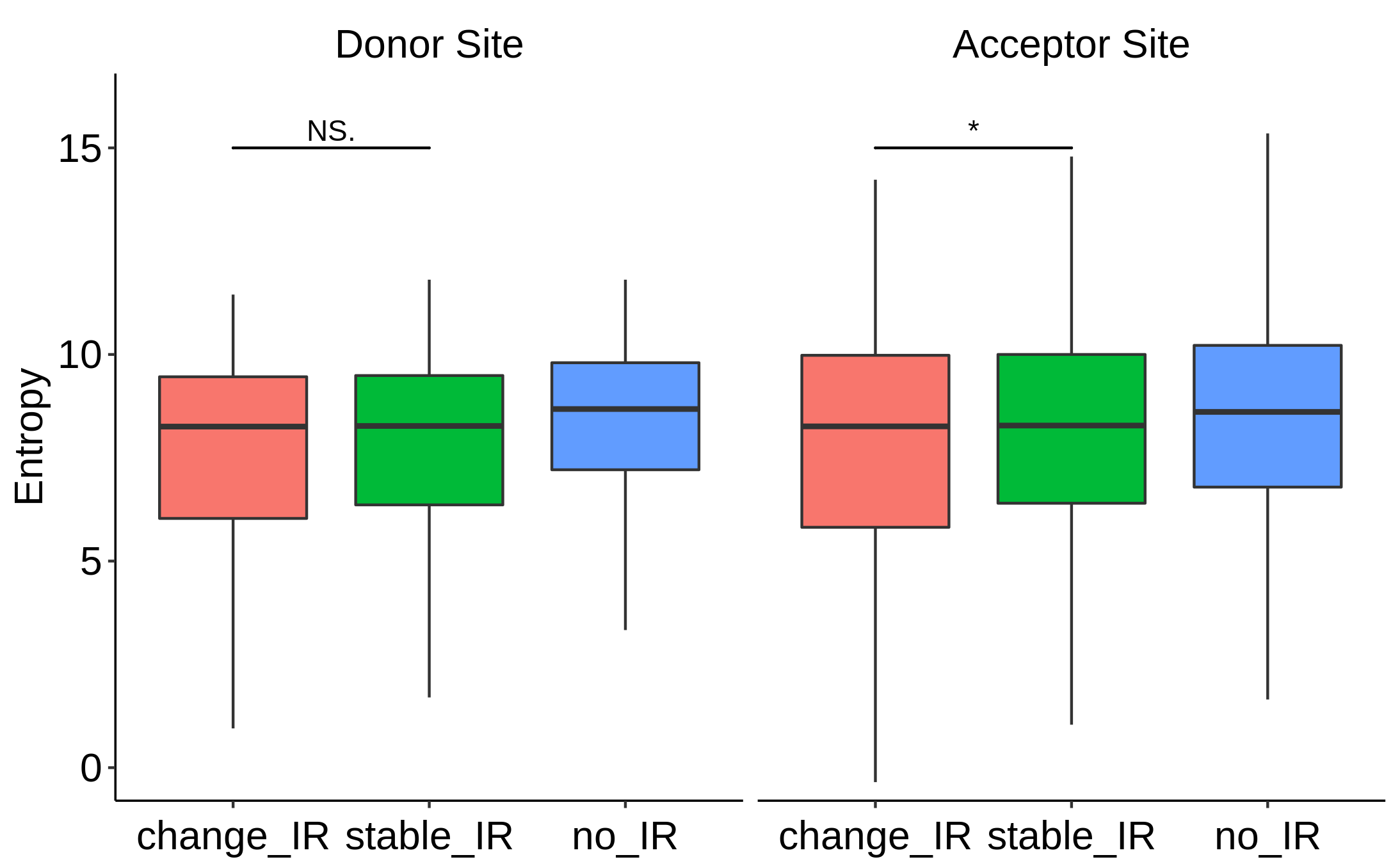


Figure S9: Comparison of splicing strength (measured using maximum entropy) of donor and acceptor splice sites for regulated (change_IR) and constitutively retained (stable_IR) introns (two-sided Wilcoxon test).

#####


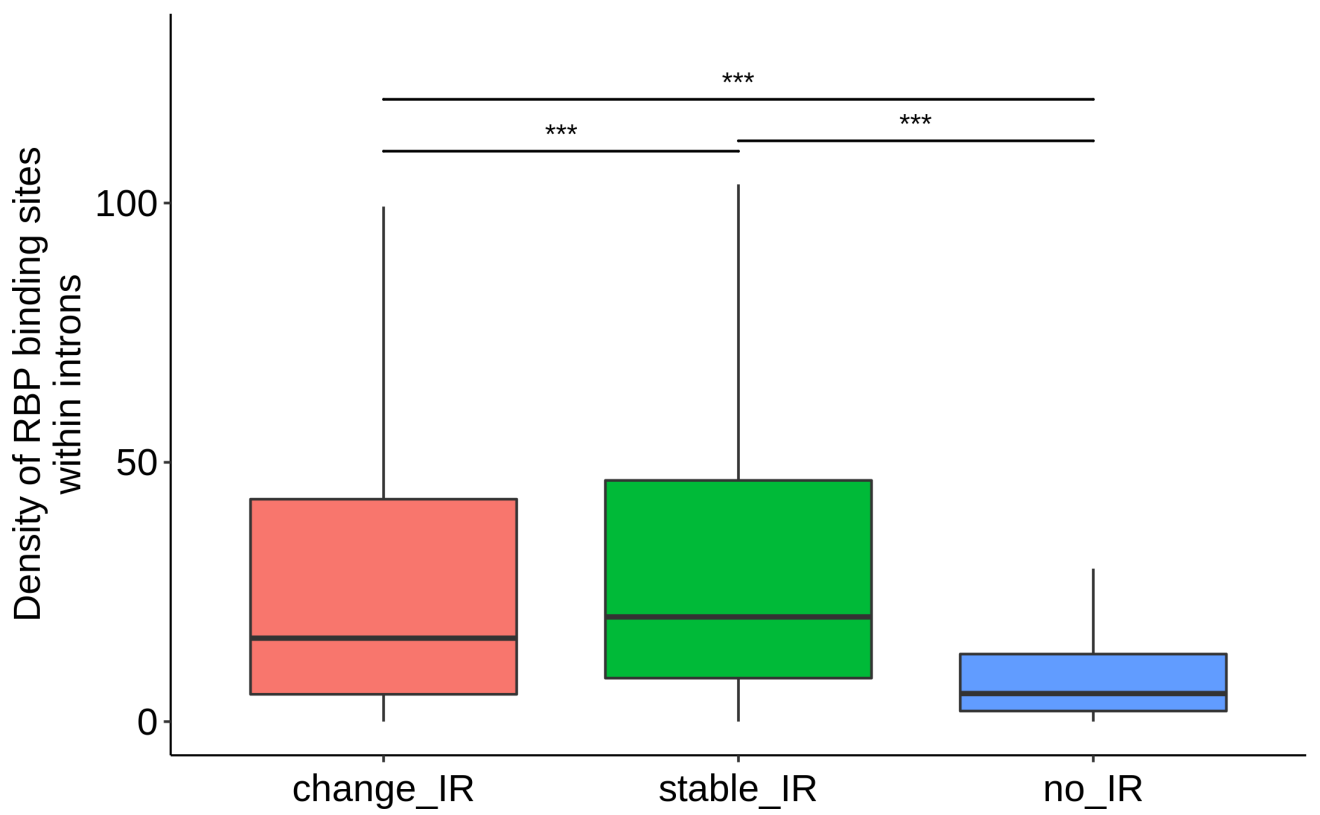


Figure S10: Density of RBP binding sites in regulated (change_IR), stably retained (stable_IR) and spliced (no_IR) introns.

#####


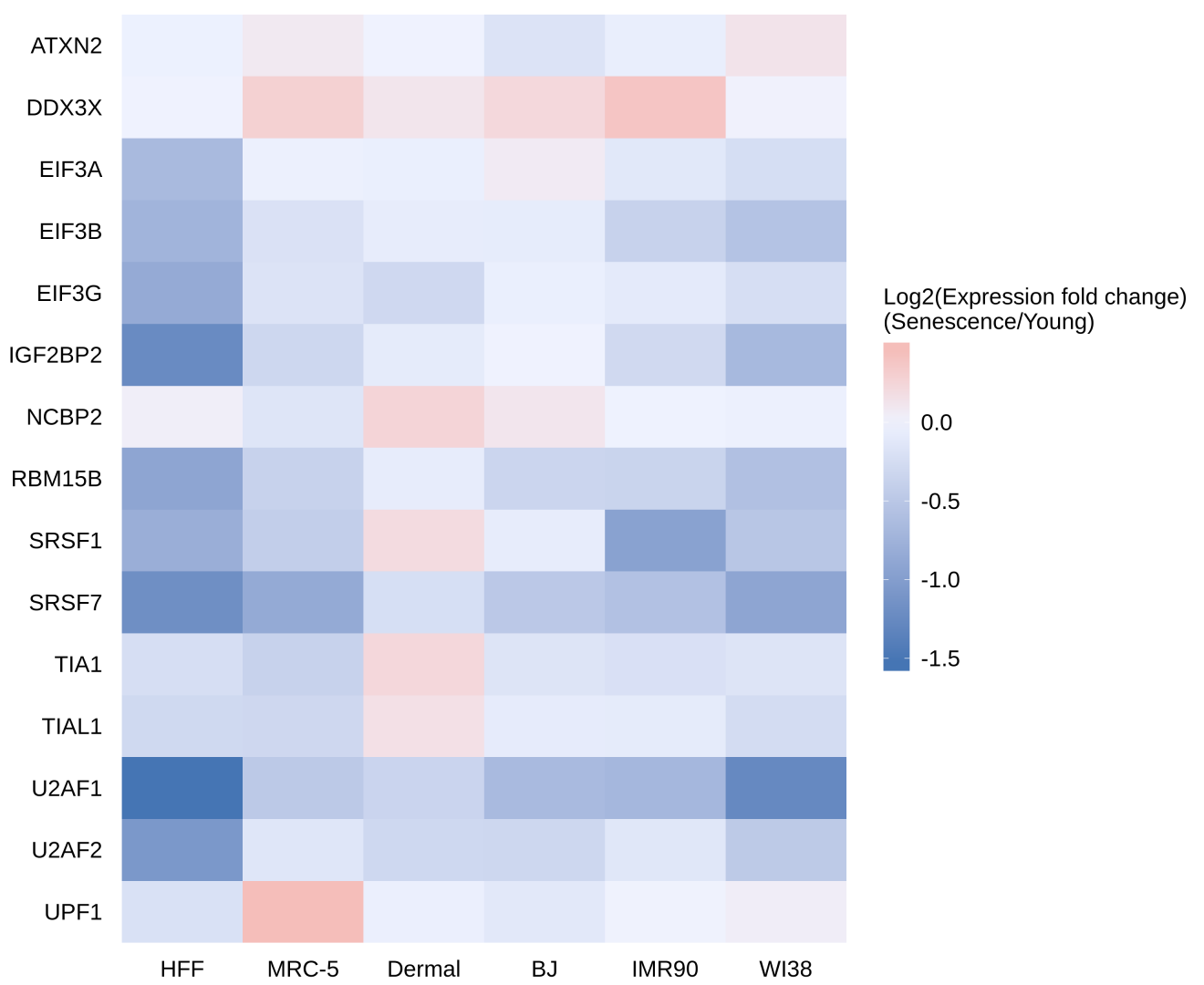


Figure S11: Heatmap showing log_2_ transformed expression fold changes of 15 candidate RBPs in multiple cellular senescence models. ‘Dermal’ denotes dermal fibroblasts derived from young and aged individuals.

#####


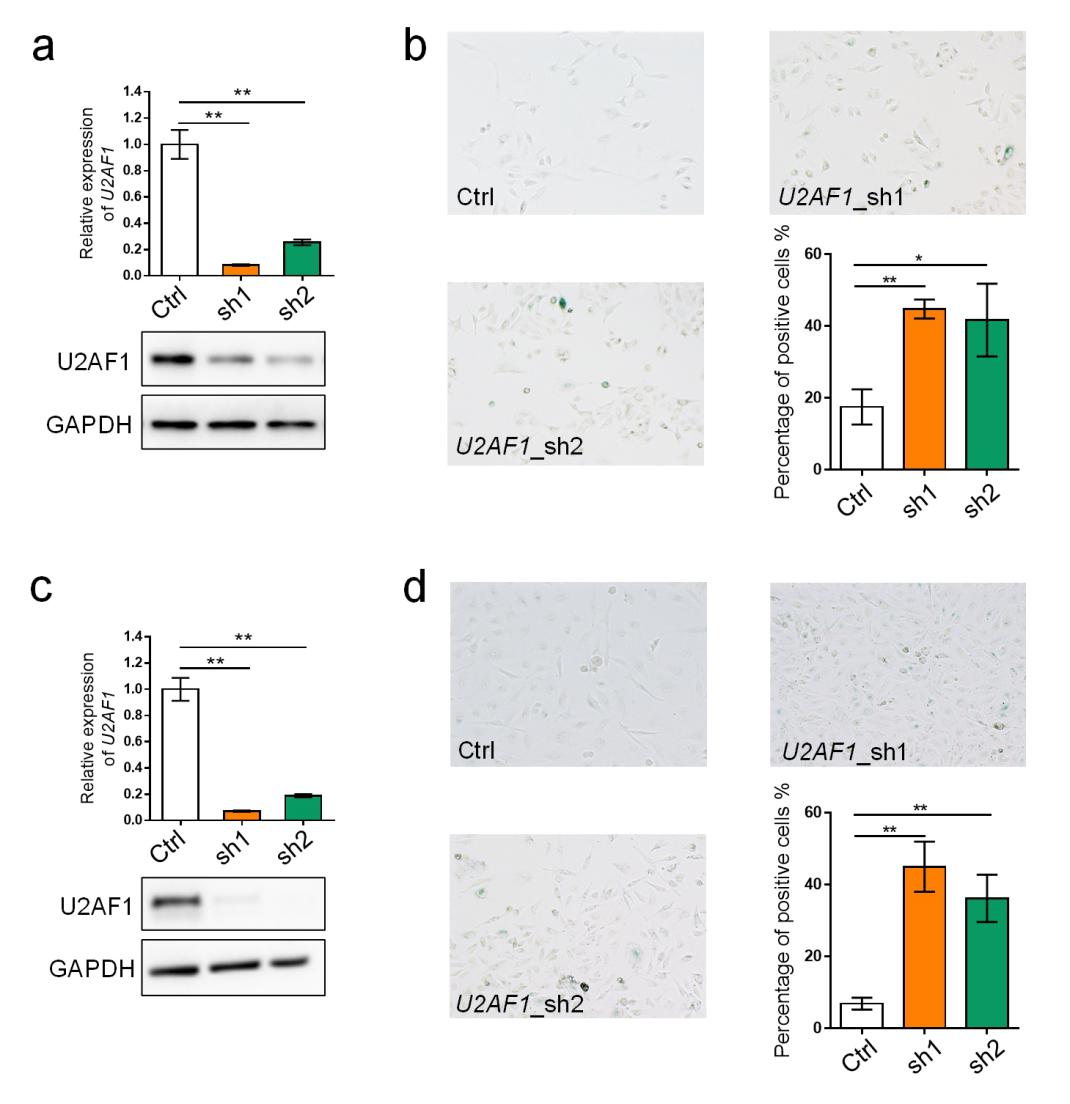


Figure S12: **Decreased U2AF1 level induces senescence in A549 and HUVEC cells.** (a) Validation of *U2AF1*-KD in A549 cell through qRT-PCR (upper panel) and Western blot (lower panel). (b) SA-β-Gal staining of *U2AF1*-KD and control (Ctrl) A549 cells. Bar chart shows the percentage of positive SA-β-Gal staining cells. (c) Validation of *U2AF1*-KD in HUVEC cell through qRT-PCR (upper panel) and Western blot (lower panel). (d) SA-β-Gal staining of *U2AF1*-KD and control (Ctrl) HUVEC cells. Bar chart shows the percentage of positive SA-β-Gal staining cells. _sh1 and _sh2 represent the two shRNAs targeting *U2AF1*. * and ** denote $P<0.05$ and $P<0.01$, respectively, two-tailed *t*-test.

#####


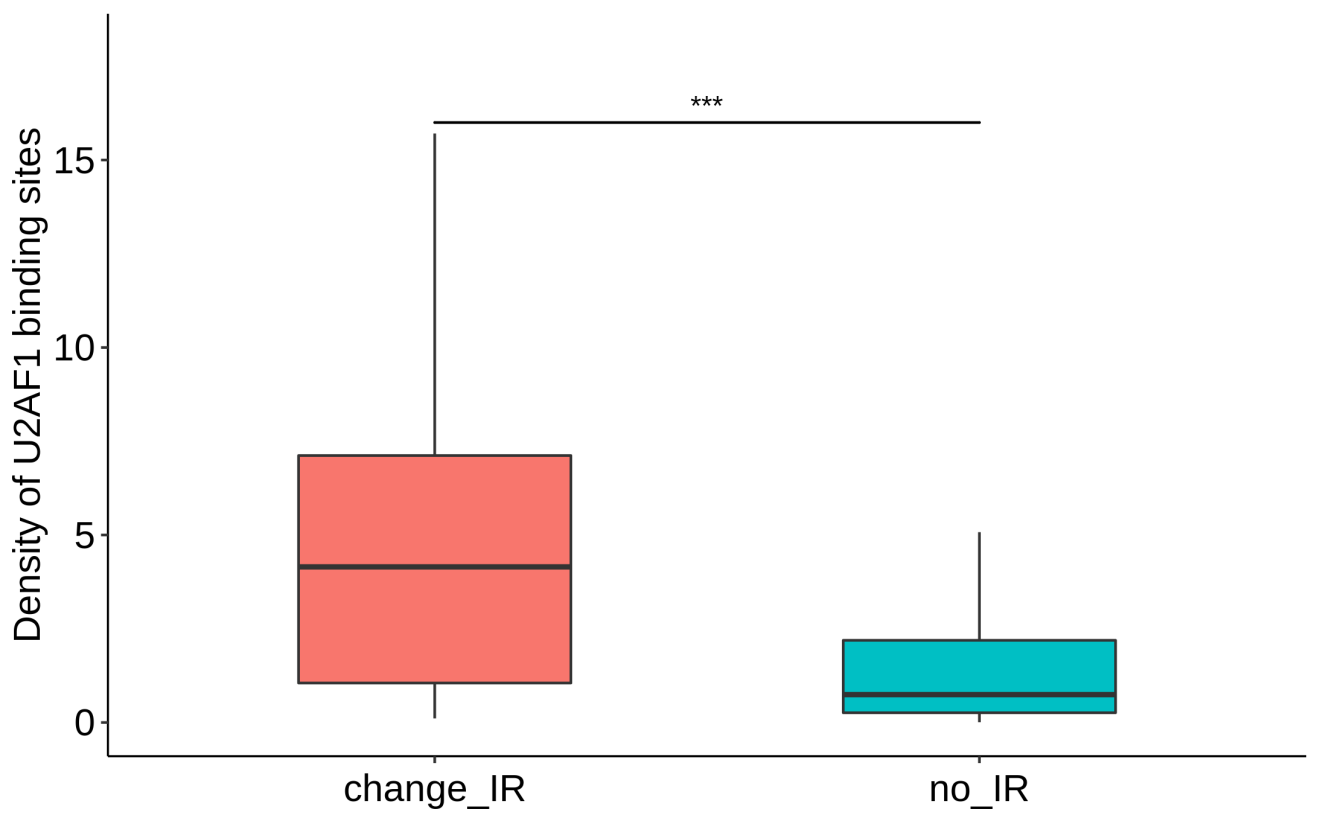


Figure S13: Boxplot showing U2AF1 binding density higher in regulated introns (change_IR) than spliced introns (no_IR) in MRC-5 cells. *** indicates $P<0.001$, one-sided Wilcoxon test.

#####


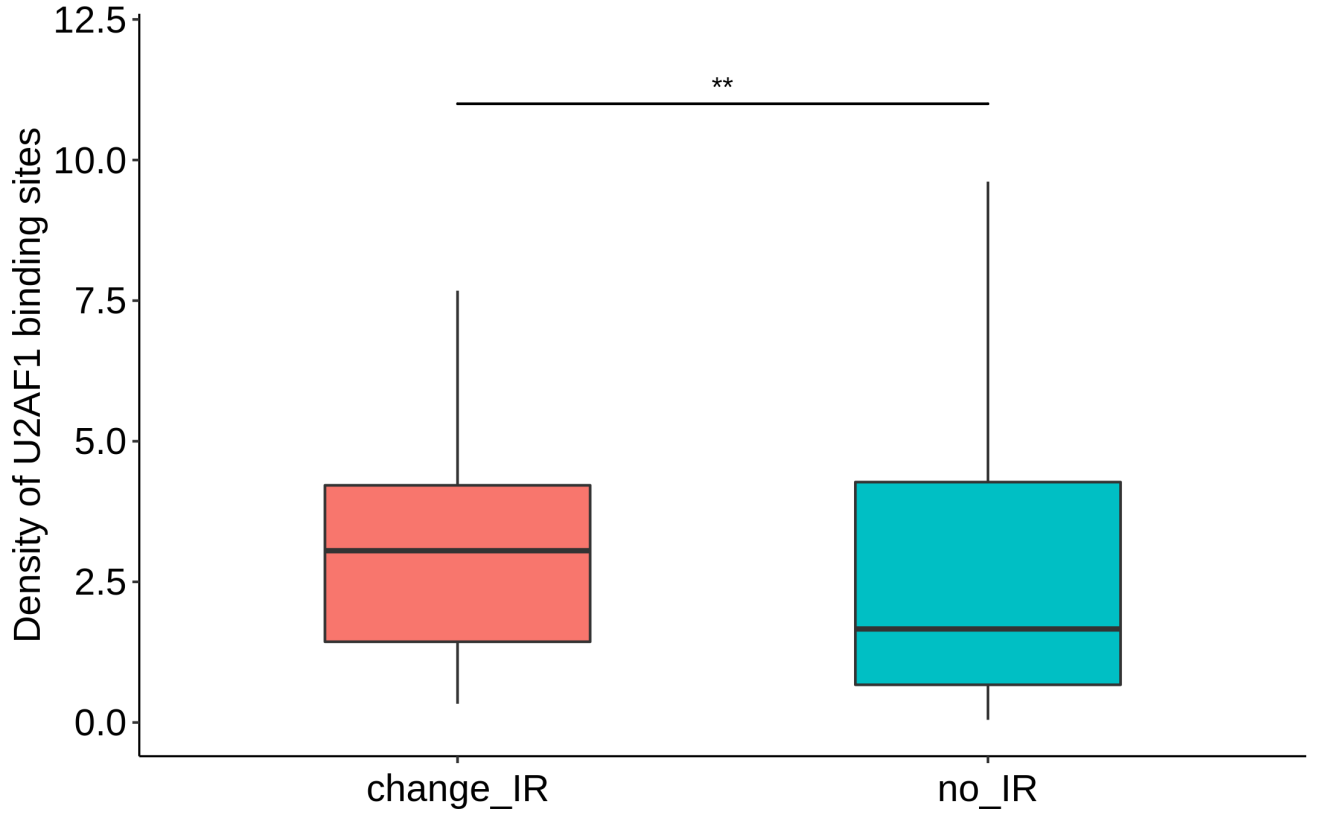


Figure S14: Boxplot showing U2AF1 binding density higher in regulated introns (change_IR) than spliced introns (no_IR) in human dermal fibroblasts derived from young and aged individuals. ** indicates $P<0.01$, one-sided Wilcoxon test.

#####


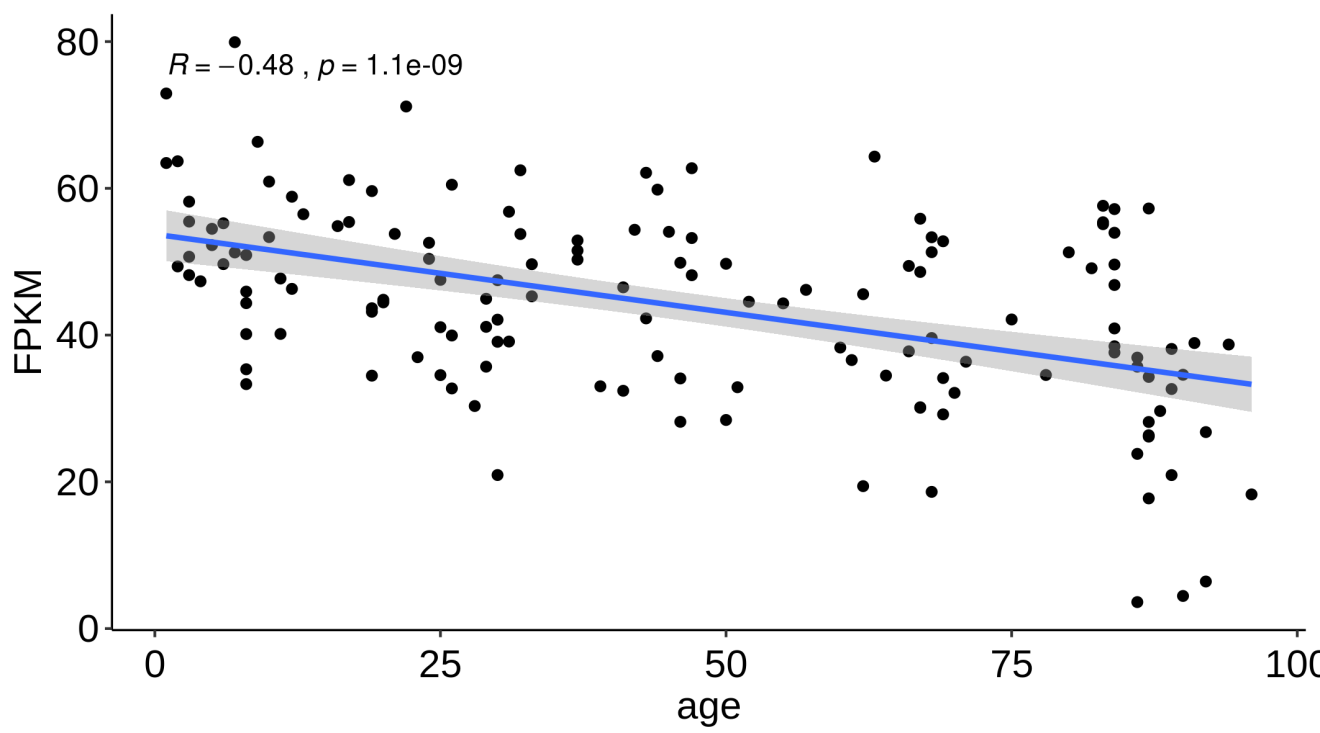


Figure S15: Scatter plot showing decreased expression trend of *U2AF1* based on RNA-seq dataset of human dermal fibroblast from young and aged individuals. Both the Pearson’s correlation coefficient and its significance were calculated by R function cor.test. The *R* value denotes the Pearson’s correlation coefficient and the *p* value denotes the significance of the result determined by two-sided *t* test. Two-sided means that the alternative hypothesis is positive or negative association between expression level of *U2AF1* and age.

#####


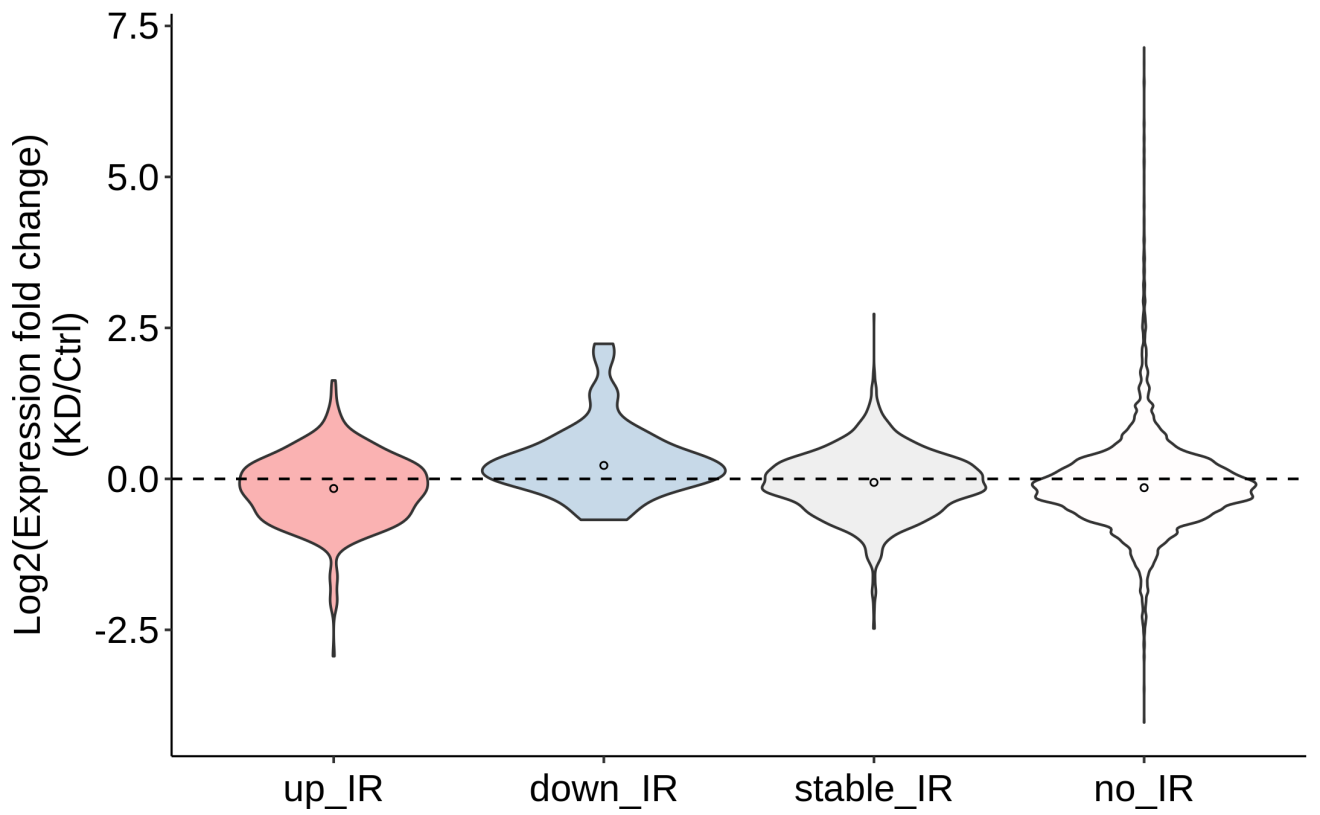


Figure S16: Violin plot showing the distribution of gene expression change between *U2AF1*-KD and control HFF cells for genes corresponding to different IR-related groups.

#####


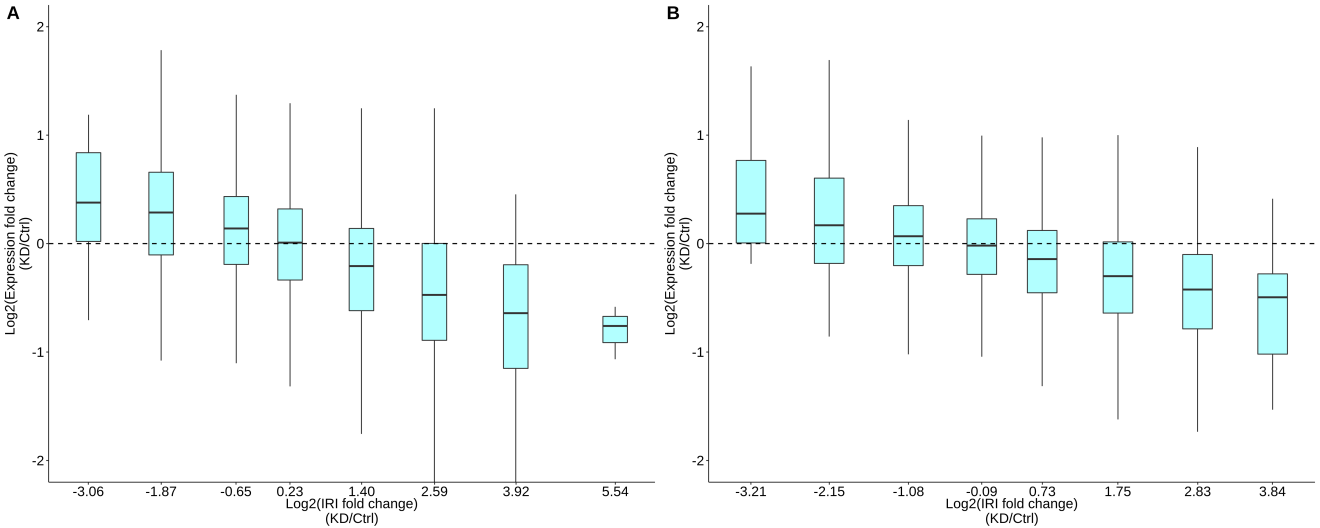


Figure S17: Boxplot showing the degree of IR changes is negatively correlated with the extent of expression fold changes for two shRNAs (A, B for sh1, sh2, respectively). Genes were divided into 8 bins based on IRI fold changes between *U2AF1*-KD HFF cells to control cells (X axis).

#####


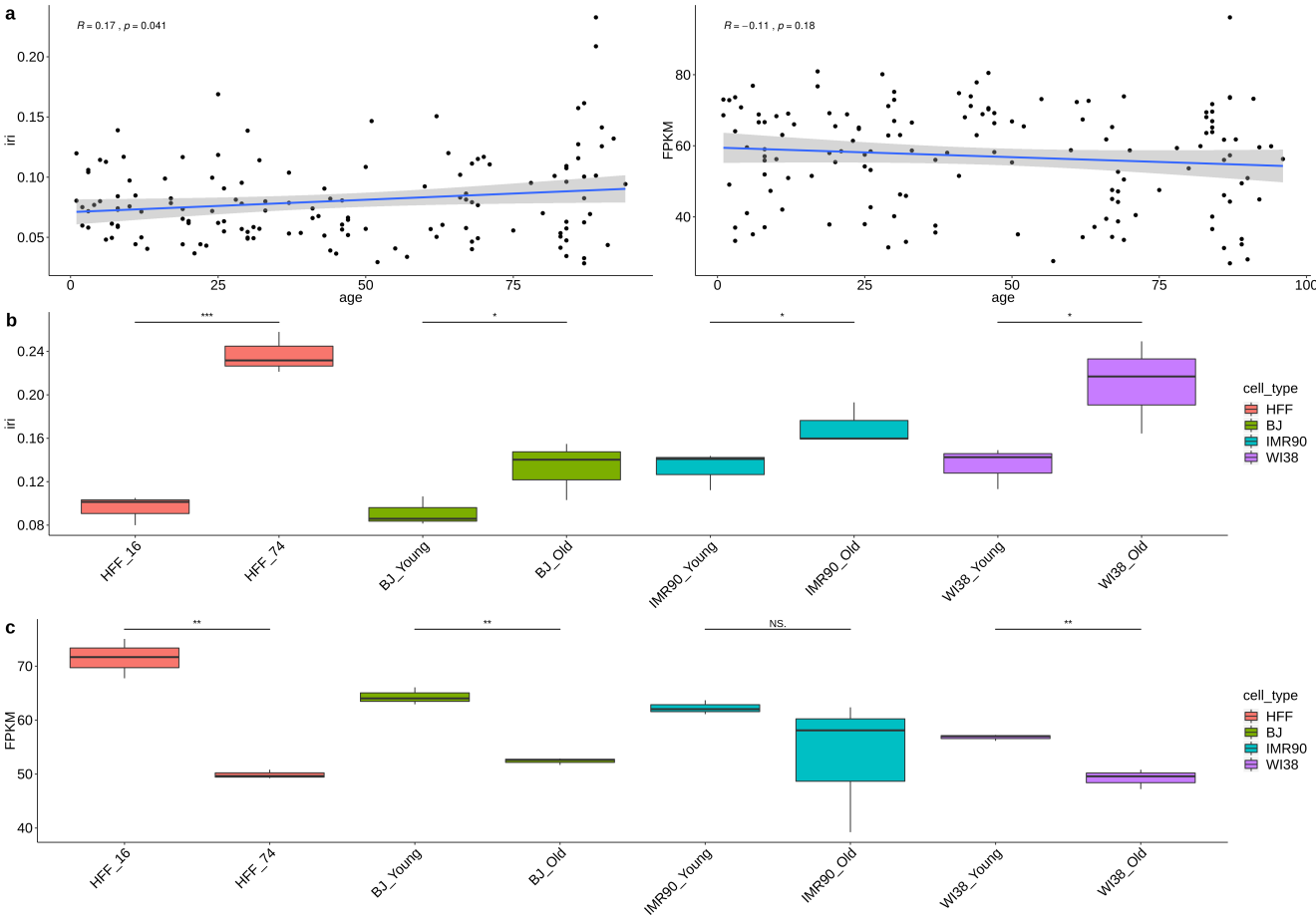


Figure S18: **Comparison of IRI and expression level of *CPNE1* in multiple senescence/aging models.** (a) Scatter plots showing IR change trend (left panel) and expression change trend (right panel) of *CPNE1* in dermal fibroblasts aging model. Both the Pearson’s correlation coefficient and its significance were calculated by R function cor.test. The *R* value denotes the Pearson’s correlation coefficient, and the *p* value denotes the significance of the result assessed by two-sided *t* test. Two-sided means that the alternative hypothesis is positive or negative association between IR or expression level of *CPNE1* and age. (b, c) Boxplots showing IR (b) and expression level (c) of *CPNE1* in multiple replicative senescence models. *, ** and *** denote $P<0.05$, $P<0.01$ and $P<0.001$, respectively, one-sided *t*-test.

#####


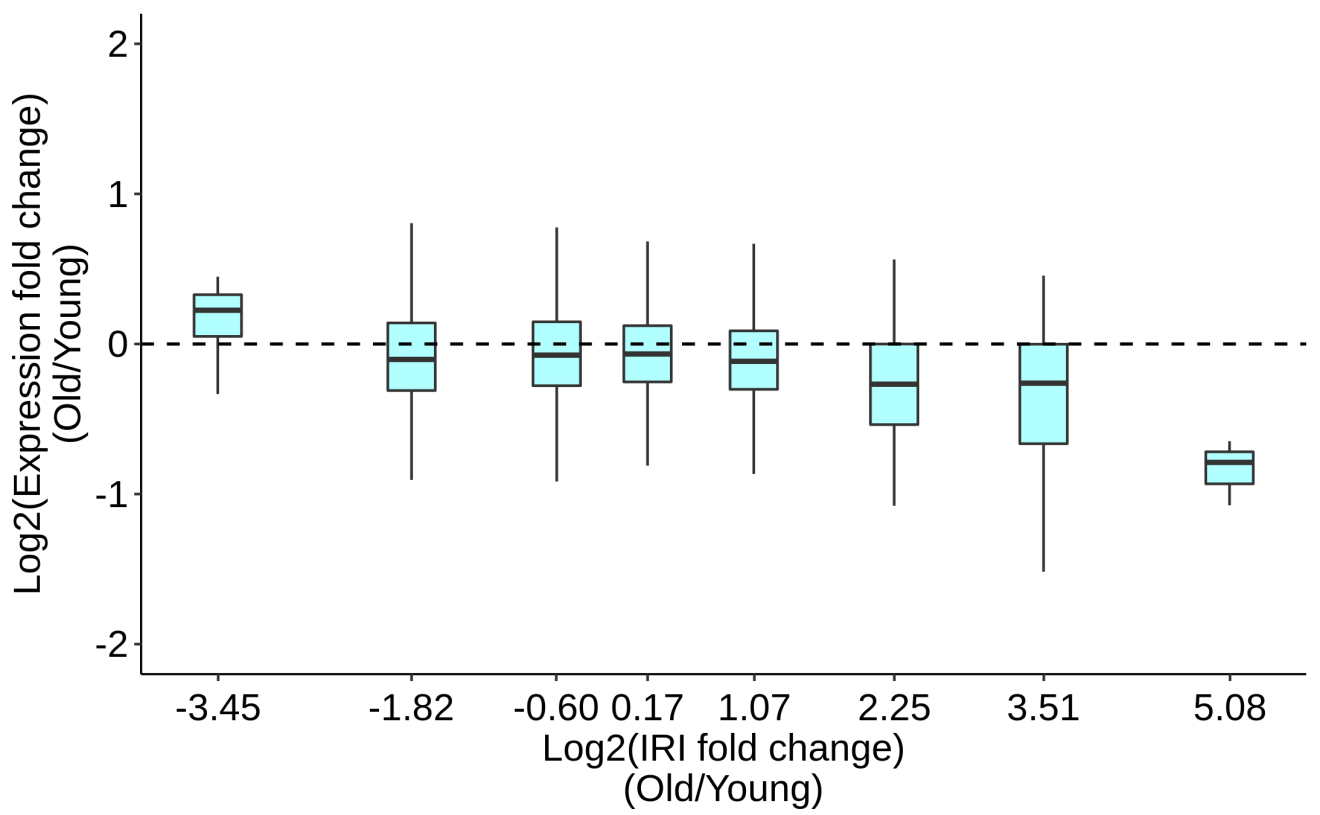


Figure S19: Boxplot showing the negative correlation between IR changes and expression alteration of corresponding genes in human dermal fibroblasts. Genes were divided into 8 bins based on IRI fold changes between old (over 80 years old) and young (20 to 40 years old) individuals.

#####


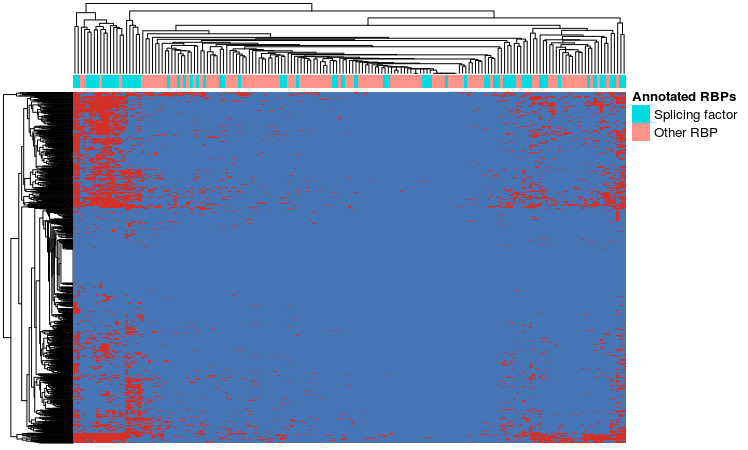


Figure S20: Heatmap showing RBP binding state of regulated introns (up_IR + down_IR). RBPs are from the POSTAR2 database. Each line stands for a retained intron and each column stands for an RBP. Splicing factors were selected by searching keywords “splicing/spliceosome” among manually annotated and reviewed human proteins from UniProtKB database. Red indicates binding while blue indicates no binding. Hierarchical clustering method was used to cluster the retained introns and RBPs.

#####


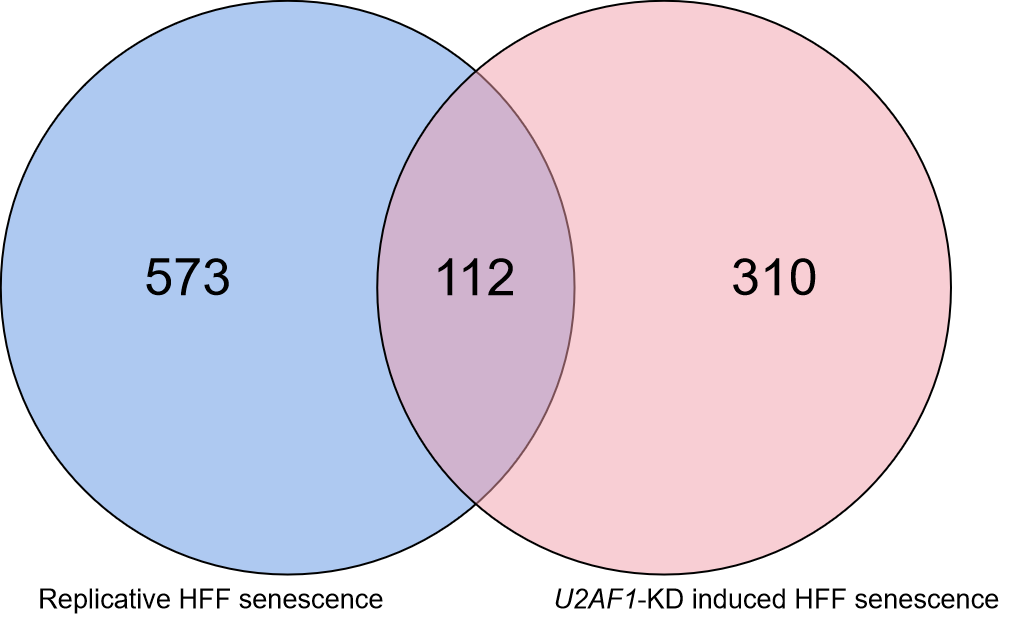


Figure S21: Venn diagram showing the relation between genes with altered IR in replicative HFF senescence and those in U2AF1-KD induced HFF senescence.

#####


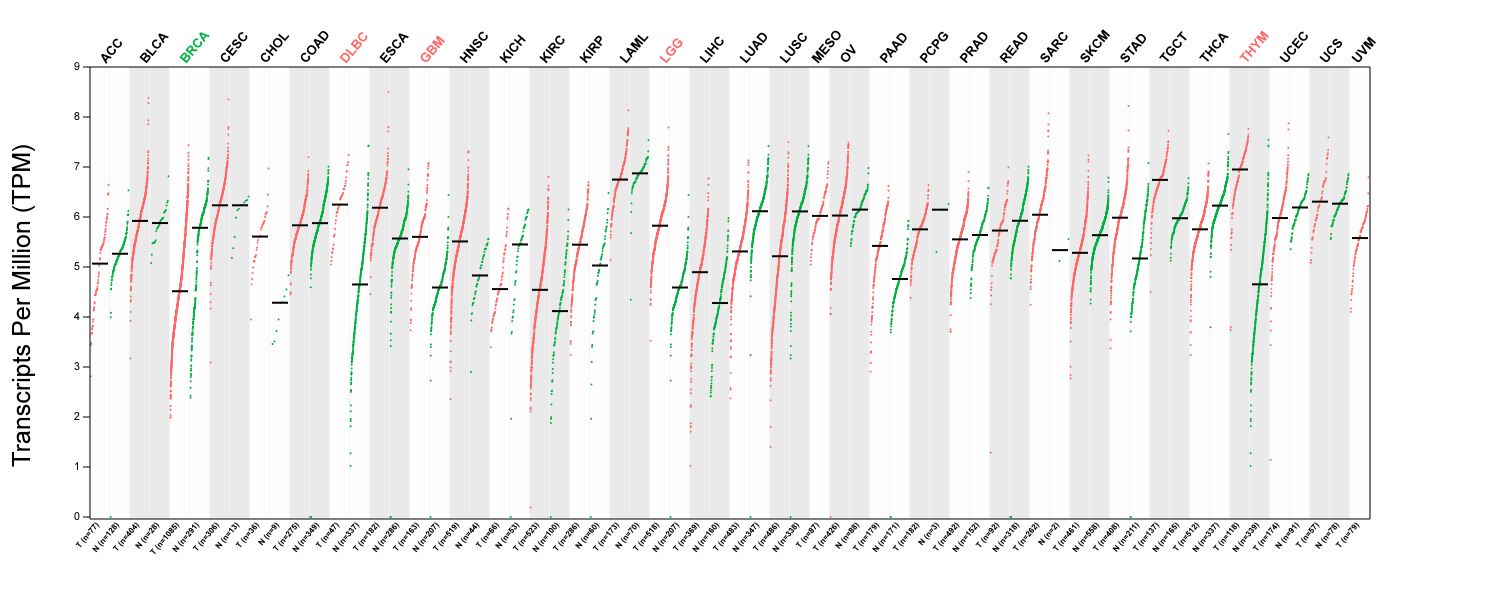


Figure S22: Dot plot showing the gene expression profile of *U2AF1* in various tumor samples and matched normal tissues. Each dot represents log_2_ transformed expression (TPM) of a sample.

#####


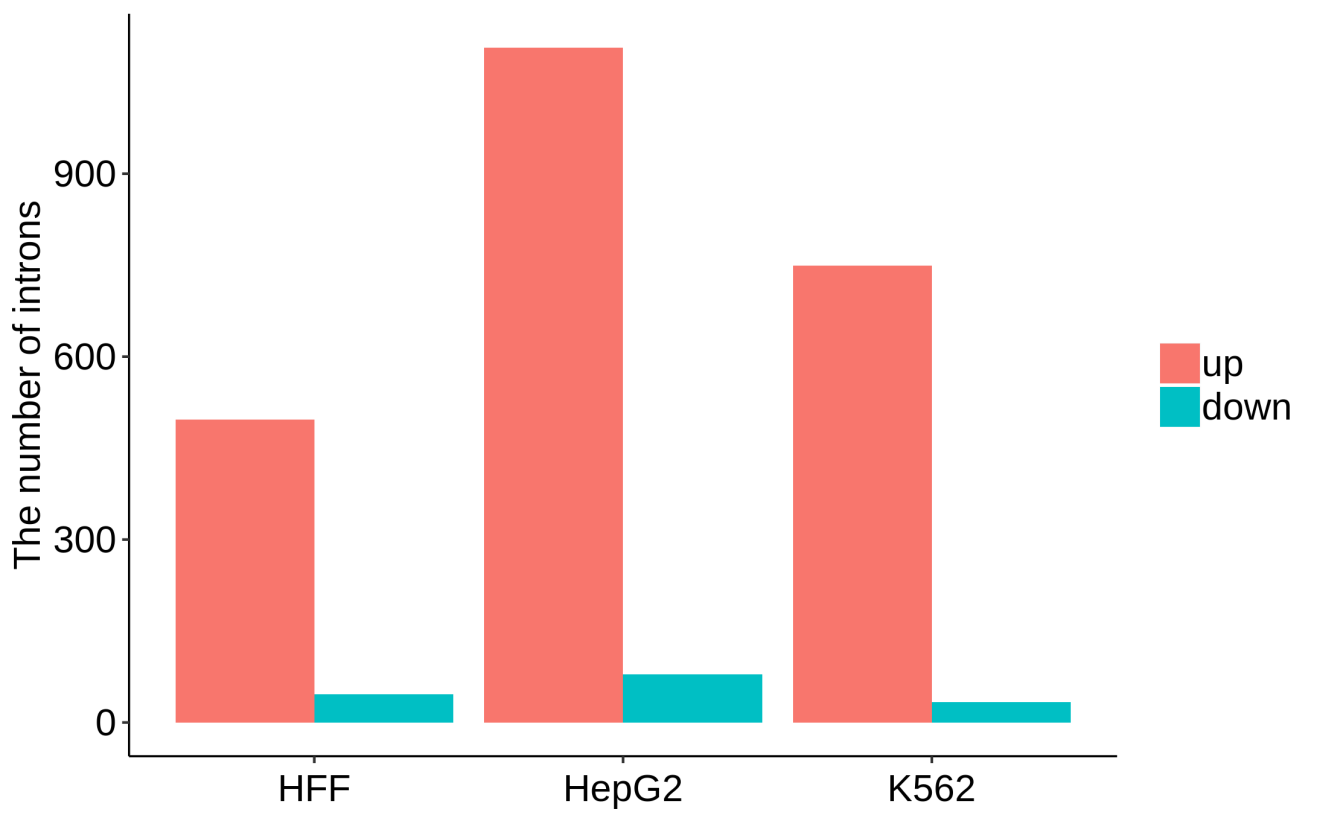


Figure S23: Barplot showing the number of up and down regulated (evaluated by IRI change) retained introns upon *U2AF1*-KD in HFF, HepG2 and K562 cells.

#####


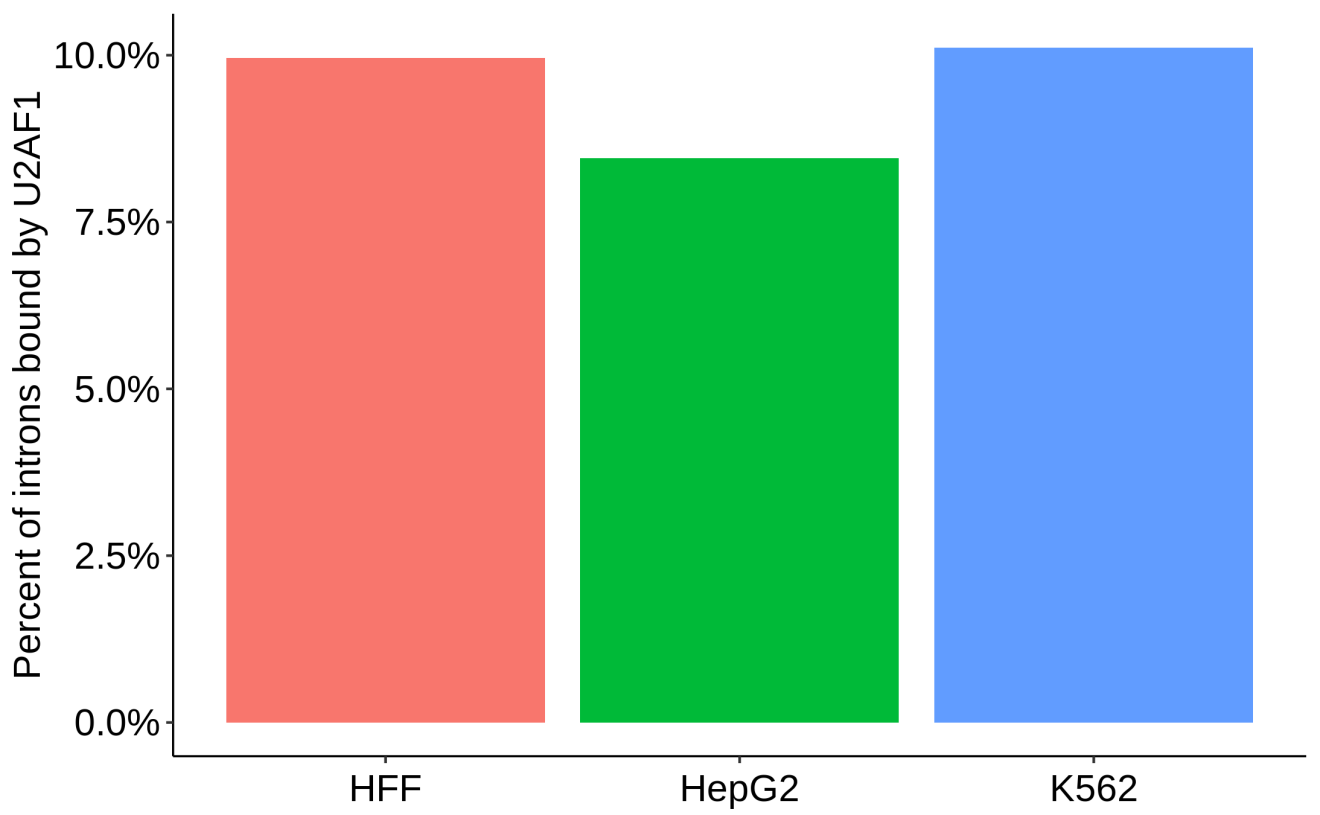


Figure S24: Barplot showing the percentage of regulated introns bound by U2AF1 in HFF, HepG2 and K562 cells.

#####


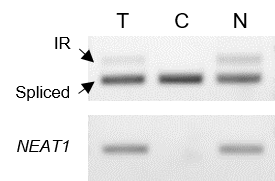


Figure S25: **IR transcripts of *CPNE1* are retained in nucleus in HFF cells.** IR and spliced transcripts of *CPNE1* are detected by RT-PCR in total cells (T), cytoplasm (C) and nucleus (N), respectively. *NEAT1*, a non-coding RNA, serves as a positive nuclear marker.

#####

## Supplemental Tables

**Table S1. Summary of IRI values for HFF senescence dataset.**

##

**Table S2. Summary of IRI values for control versus *U2AF1* knockdown cells.**

##

**Table S3. Candidate introns where U2AF1 mediates IR to senescence in HFF cells.**

##

**Table S4. The numbers of total and senescence-associated genes with splicing events for each AS type upon *U2AF1* knockdown.**

##

**Table S5. Sequences of shRNAs and primers used in this study.**
